# Identification of Reference Genes for Real-Time Quantitative PCR Experiments in the Liverwort *Marchantia polymorpha*

**DOI:** 10.1371/journal.pone.0118678

**Published:** 2015-03-23

**Authors:** Denis Saint-Marcoux, Hélène Proust, Liam Dolan, Jane A. Langdale

**Affiliations:** Department of Plant Sciences, University of Oxford, Oxford, United Kingdom; Universidade Federal do Rio Grande do Sul, BRAZIL

## Abstract

Real-time quantitative polymerase chain reaction (qPCR) has become widely used as a method to compare gene transcript levels across different conditions. However, selection of suitable reference genes to normalize qPCR data is required for accurate transcript level analysis. Recently, *Marchantia polymorpha* has been adopted as a model for the study of liverwort development and land plant evolution. Identification of appropriate reference genes has therefore become a necessity for gene expression studies. In this study, transcript levels of eleven candidate reference genes have been analyzed across a range of biological contexts that encompass abiotic stress, hormone treatment and different developmental stages. The consistency of transcript levels was assessed using both geNorm and NormFinder algorithms, and a consensus ranking of the different candidate genes was then obtained. *MpAPT* and *MpACT* showed relatively constant transcript levels across all conditions tested whereas the transcript levels of other candidate genes were clearly influenced by experimental conditions. By analyzing transcript levels of phosphate and nitrate starvation reporter genes, we confirmed that *MpAPT* and *MpACT* are suitable reference genes in *M*. *polymorpha* and also demonstrated that normalization with an inappropriate gene can lead to erroneous analysis of qPCR data.

## Introduction

Real-time quantitative polymerase chain reaction (qPCR) is a sensitive and reproducible technique that is used to measure and compare transcript accumulation levels for genes in many organisms. Correct interpretation of qPCR results, however, is subject to an understanding of several parameters (e.g. the amount and integrity of initial RNA, variations between batches of reagents, and intrinsic stochastic variability in the biochemical reactions). In an attempt to standardize procedures and thus allow comparability between experiments, guidelines establishing a checklist for qPCR experiments were published several years ago [1–3]. Notably, the use of an appropriate normalization strategy is perhaps the most important point to consider for accurate transcript level comparison, because normalization can compensate for experimental variations between different samples. Normalization in qPCR experiments is most commonly carried out by comparing transcript levels of the gene of interest with transcript levels of a ‘constitutively expressed’ reference gene. As such, qPCR experiments can only accurately report transcript levels for a gene of interest, if there are suitable reference genes available for use with the biological samples under investigation [4,5].

*Marchantia polymorpha* is a liverwort that belongs to the early divergent bryophyte grade of land plants [6,7]. Although the phylogenetic relationship between the bryophyte lineages of liverworts, mosses and hornworts is still debated, the position of liverworts as one of (if not) the earliest land plant lineage(s) is undisputed [8]. As such, studies of liverwort biology are important for understanding the evolution of development processes in land plants. Over the last few years, *M*. *polymorpha* has become the liverwort species of choice, not least because it is easy to propagate, mutate and genetically transform [9–11]. The adoption of *M*. *polymorpha* as a model [12–14], has led to the need to develop quantitative methods for analyzing mRNA levels. As such, reference genes for which transcript levels are relatively constant across a range of routine conditions must now be identified. The identification of suitable genes for normalization of qPCR analyses in *M*. *polymorpha* will facilitate gene expression studies in a representative of one of the earliest land plant lineages.

Specialized tools have been developed over the years to help identify good reference genes for qPCR analyses [15–18], and a number of studies reporting suitable genes have been published in a diverse range of organisms [4,5,18–28]. Notably, studies suggested that commonly used reference genes such as *ACTIN*, *TUBULIN*, *UBIQUITIN* or *GLYCERALDEHYDE-3-PHOSPHATE DEHYDROGENASE* are not the most appropriate [4,5,20,24,25], and a number of studies have suggested that genes such as *SAND* [20], *EUKARYOTIC ELONGATION FACTOR 5* [28], or *CULLIN* [24] are better alternatives. Here, we report the analysis of transcript levels for eleven genes in *M*. *polymorpha* across different growth conditions, a range of developmental stages, and under a variety of abiotic stress and hormone treatments. We identify *MpAPT* and *MpACT* as a good pair of reference genes for normalization of qPCR assays in *M*. *polymorpha* and demonstrate that *MpGAPC1*, *MpTUB8* and *MpUBQ10* are unsuitable. Finally, using phosphate and nitrate starvation reporter genes, we exemplify how choosing suitable reference genes is crucial for the accurate analysis of qPCR data.

## Materials and Methods

### Plant material and culture conditions

Gemmae from female *Marchantia polymorpha* (accession Takaragaike-2) [11] were propagated on Petri dishes filled with ½ Gamborg’s medium pH 5.6 (# G0210, Duchefa) supplemented with 1% agar, or in pots filled with a soil-based compost (Levingston M2) under a 16h light:8h dark photoperiod, respectively at 23°C and a light intensity of 56 μE.m^−2^.s^−1^, or at 18°C and a light intensity of 150 μE.m^−2^.s^−1^. Male and female sexual organs were obtained from 2-month-old plants (accession Takaragaike-1 and Takaragaike-2 respectively) cultivated in soil conditions supplemented with far-red light [29,30].

For cold treatment, 17 day old plants were transferred to 4°C for 24 hours. Hormone treatments were carried out on ½ Gamborg’s supplemented with, abscisic acid (ABA), 1-naphthaleneacetic acid (NAA) or GR24 at a final concentration of 2 μM, 750 nM and 1 μM respectively. Hormones were initially dissolved in 100% acetone then dilutions to 1/10000^th^ were prepared to supplement the medium. Medium for starvation experiments was prepared by modulating phosphate or nitrate concentration in ½ Johnson’s medium [31]. Low phosphate medium was prepared by adding only 1 mM of (NH_4_)_2_SO_4_ instead of 1 mM of NH_4_H_2_PO_4_. For both low and high nitrate media, 2 mM of CaCl_2_ was added to the medium instead of 2 mM of Ca(NO_3_). Low nitrate medium was prepared by adding 1 mM KNO_3_ instead of 3 mM KNO_3_, and 2 mM of KH_2_PO_4_ as phosphate source. High nitrate medium was prepared by adding 5 mM of NH_4_NO_3_, and 2 mM of NH_4_H_2_PO_4_ as phosphate source. For 24 hours treatments (with the exception of cold treatment), plants were grown on cellophane disks (AA Package Ltd.) placed on top of untreated medium. Cellophane disks were then transferred onto untreated (control) or treated (induction) medium.

### RNA extraction, cDNA synthesis and qPCR

Total RNA was extracted from ground frozen tissue with the RNeasy plant mini kit (#74904, Qiagen). On-column DNase I treatment was performed using RNase-free DNase I (#79254, Qiagen) according to the manufacturer’s recommendations. Total RNA samples were quantified using a Nanodrop ND-1000 spectrophotometer. RNA integrity was checked using a 2100 BioAnalyzer (Agilent Technologies) and RNA-Nano chips (#5067-1511, Agilent Technologies). The RIN (RNA integrity number) provided by the BioAnalyzer software ranges from 1 (corresponding to a very degraded sample) to 10 (for a mostly intact sample).

cDNA was synthesized from 1 μg of total RNA. Reverse transcription was performed in a 20 μl volume with 2 μM of poly-dT_17_ primer and 5 U of superscript III reverse transcriptase (#18064, Fermentas) according to the manufacturer’s protocol. cDNA was then diluted 10 times by adding 180 μl nuclease-free water.

Amplification experiments were carried out using a 7300 Applied Biosystem thermocycler. Reactions were performed in 10 μL volumes containing 5 μL 2X SYBR-green mastermix (#4309155, Applied Biosystem), 500 nM forward and reverse primers, and 4 μl diluted cDNA (equivalent to 20 ng of reverse transcribed total RNA). A two-step cycle composed of denaturation at 94°C for 15 seconds followed by hybridization/elongation at 60°C for 60 seconds, was repeated 40 times and then followed by a dissociation step. Three technical replicates were performed for each reaction. Genomic contamination was determined by direct amplification of 20 ng total RNA for 50 cycles.

### Data analysis

Fluorescence data were first manually checked for any substantial variation between technical replicates and any outlying values were discarded. Fluorescence data were then exported and transformed using a Perl script into a format suitable for analysis using LinRegPCR v2012.0 [32]. Briefly, LinRegPCR determines mean reaction efficiency (E_m_) for any specific primer pair, by linearly regressing fluorescence data from several amplification reactions. Because mean efficiencies are calculated in this way, where possible, reactions were grouped in qPCR plates by amplicon rather than by treatment/growth condition. Using the mean efficiency and the window of linearity of the reaction, LinRegPCR computes a N_0_ value which reflects the initial amount of template in the reaction mix. The computed N_0_ values from the technical replicates were then averaged. Genomic contamination was taken into account by subtracting N_0_ values obtained from *MpEF1α* amplified from total RNA. Contamination was insignificant in all cases, with N_0_ values from genomic amplifications at least two orders of magnitude lower than N_0_ from cDNA amplifications.

The extent of variation in transcript levels across samples was determined using the software GenEx version 6 (http://www.biomcc.com/genex-software.html), including geNorm [16] and NormFinder [15] algorithms. N_0_ values from three biological replicates were first averaged and then normalized by the highest averaged N_0_ value for a given gene. Normalized N_0_ were thus rendered equivalent to relative quantification data classically obtained by the ∆Ct method and suitable to be used in GenEx (see *Equation* 1 adapted from Ruijeter et al. [32]).

$$N_{0}/N_{0}{}_{\;\max}=E_{m}{}^{Ct_{\;\max}-Ct}$$ Equation1

Stability values were produced by geNorm and NormFinder algorithms (the lower the value, the more stable the gene transcript level). For a given gene, geNorm computes the standard deviation of transcript level ratios between the gene and another gene in all tested conditions. The stability value is then equal to the average of the standard deviations computed for every pairwise comparison of the gene. In contrast, NormFinder relies on a mathematical model to describe gene transcript level variations for a given tested condition and between all tested conditions. The sum of both terms produces a stability value.

Stability values were transformed into z-scores (*z*) using *Equation* 2.

$$z=\left(S_{n}-{\bar{X}}_{S}\right)/\delta_{S}$$ Equation2

with *S_n_* the stability value of a given gene n, ${\bar{x}}_{s}$ and *δ_S_* the mean and the standard deviation of the stability values respectively.

## Results

### Identification of candidate reference genes

To select putative reference genes for qPCR in *M*. *polymorpha*, we conducted a survey of genes used in other plant species [4,5,17,19–28]. 11 candidate genes were selected and orthologous sequences were identified in a *M*. *polymorpha* thallus transcriptome (Giulia Morieri, Hélène Proust & Steve Kelly, unpublished) using *Arabidopsis* gene sequences and reciprocal best BLAST [33] (**Table 1**). Proteins encoded by the selected genes play roles in genome organization and expression (EF1α, ELONGATION FACTOR 1α; ELF5, EUKARYOTIC ELONGATION FACTOR 5A-1; APT, ADENINE PHOSPHORIBOSYL TRANSFERASE; H3, HISTONE 3), cytoskeleton formation (TUB8, TUBULIN BETA CHAIN 8; ACT, ACTIN 7), protein modification and degradation (CUL, CULLIN 1; UBQ10, POLYUBIQUITIN 10; PEX, PEROXIN 4), glycolysis (GAPC1, GLYCERALDEHYDE-3-PHOSPHATE DEHYDROGENASE C SUBUNIT 1) and vesicle trafficking (SAND).

**Table 1:** qPCR candidate reference genes in *M. polymorpha.*

**Gene name**	**Best hit in uniprot library**	**Primer sequences (Forward / Reverse)**	**PCR efficiency**	**Amplicon size (bp)**
*MpAPT*	ADENINE PHOSPHORIBOSYL TRANSFERASE 3	CGAAAGCCCAAGAAGCTACC / GTACCCCCGGTTGCAATAAG	1.870	146
*MpACT*	ACTIN 7	AGGCATCTGGTATCCACGAG / ACATGGTCGTTCCTCCAGAC	1.908	108
*MpPEX*	PEROXIN 4	CAGTCAGTTTGCCGTGCTG / GATTGTCCCCCGATCGTAAC	1.929	100
*MpUBQ10*	POLYUBIQUITIN 10	TGAAGGCCAAGATTCAGGAC / ACGAAGCACCAAATGGAGAG	1.883	140
*MpCUL*	CULLIN 1	AGGATGTGGACAAGGATAGACG / GTTGATGTGGCAACACCTTG	1.905	84
*MpELF5*	EUKARYOTIC ELONGATION FACTOR 5A-1	AGGTTTCCACCTCCAAGACC / AACGACCTCAGGGACATCAC	1.918	131
*MpEF1α*	ELONGATION FACTOR 1-ALPHA	CCGAGATCCTGACCAAGG / GAGGTGGGTACTCAGCGAAG	1.921	144
*MpTUB8*	TUBULIN BETA 8	ATCCCGACAGAATGATGCTC / ATTCATCGGCGTTCTCTACG	1.888	120
*MpH3*	HISTONE 3	ACTGATTTGCGGTTCCAGAG / CATAATCGTCACACGCTTGG	1.921	123
*MpGAPC1*	GLYCERALDEHYDE-3-PHOSPHATE DEHYDROGENASE C SUBUNIT 1	GTTCACCACCAAGGACAAGG / CTCGTTCACTCCCATGCAG	1.930	109
*MpSAND*	SAND protein (At2g28390)	GTTGATGTGTGGCACAAAGG / CAGGCATACGGGAGAAAATG	1.969	142

### Quantification of transcript levels in different biological contexts

To obtain a set of reference genes that is suitable for use across a broad range of conditions, transcript levels were quantified in 22 biological contexts regrouping different tissue types and plants subjected to different treatments (**Table 2**). The range of developmental stages included whole plants of different ages (17 days and 24 days old) grown in sterile (Gamborg’s) or soil conditions, plus gemmae (vegetative propagules), antheridiophores (male reproductive structures), and archegoniophores (female reproductive structures). Treatments included abiotic stresses such as nitrogen and phosphate starvation for 17 days or 24 hours (nitrogen only) or 24 hours cold exposure; and hormone treatments where plants were either grown on NAA and ABA-containing media or exposed to NAA, ABA and GR24 (a synthetic strigolactone) for 24 hours.

**Table 2:** Tissue-types, growth conditions, and exogenous treatments tested (biological contexts).

**Subgroup**	**Tissue**	**Condition**	**Age (days)**
***Development***			
	Whole plant	½ Gamborg’s	17
	Whole plant	½ Gamborg’s	24
	Whole plant	Soil	17
	Whole plant	Soil	24
	Gemmae	Soil	0
	Antheridiophore	Soil	30
	Archegoniophore	Soil	30
***Abiotic Stress***			
	Whole plant	Johnson’s HP	17
	Whole plant	Johnson’s LP	17
	Whole plant	Johnson’s HN	17
	Whole plant	Johnson’s LN	17
	Whole plant	Johnson’s HN + 24h Johnson’s HN	18
	Whole plant	Johnson’s HN + 24h Johnson’s LN	18
	Whole plant	½ Gamborg’s + 24h cold	18
***Hormom treatments***			
	Whole plant	½ Gamborg’s mock*	17
	Whole plant	½ Gamborg’s ABA 2uM	17
	Whole plant	½ Gamborg’s mock*	17
	Whole plant	½ Gamborg’s NAA 750nM	17
	Whole plant	½ Gamborg’s + 24h mock*	18
	Whole plant	½ Gamborg’s + 24h ABA 2uM	18
	Whole plant	½ Gamborg’s + 24h NAA 2uM	18
	Whole plant	½ Gamborg’s + 24h GR24 1uM	18

*acetone 1/10000^e^

Where applicable, phenotypic differences between normal and treated plants were recorded (**S1 Fig.**). Plants cultivated for 17 days on ABA (**S1B Fig.**) showed a notable size reduction compared to untreated plants (**S1A Fig.**). Similarly, when cultivated on NAA (**S1D Fig.**), plant growth was drastically impaired compared to untreated controls (**S1C Fig.**). The observed ABA– and NAA–induced phenotypes are consistent with published reports [13,34]. Minimal differences were observed between growth on low (**S1F Fig.**) versus high (**S1E Fig.**) phosphate levels for 17 days, but nitrogen starvation (**S1H Fig.**) caused a severe reduction in growth compared to growth on high nitrate levels (**S1G Fig.**). Nitrogen starvation for just 24 hours did not lead to visible growth perturbations (not shown).

qPCR amplifications were carried out using RNA extracted from plants from the 22 biological contexts (**Table 2**) and the entire experiment was replicated three times. All RNA samples showed a RIN > 7 (**S1 Table**), which is sufficient integrity for qPCR experiments [35]. cDNA was synthesized from total RNA extracts to perform qPCR reactions. Primers were designed for the 11 candidate reference genes using the Primer3Plus software [36]. To minimize amplification bias, amplicons were designed to be 80 to 150 nucleotides in length and were located in the 3’ part of the gene. Primer specificity was first checked by PCR and subsequent gel electrophoresis (not shown). The specificity of each qPCR reaction was then checked by performing a dissociation step. For each pair of primers, the dissociation curve showed a unique peak of fluorescence, indicating that a single DNA fragment was amplified during the PCR reaction (**S2 Fig.**—a few reactions are shown superimposed).

**Fig 1:**
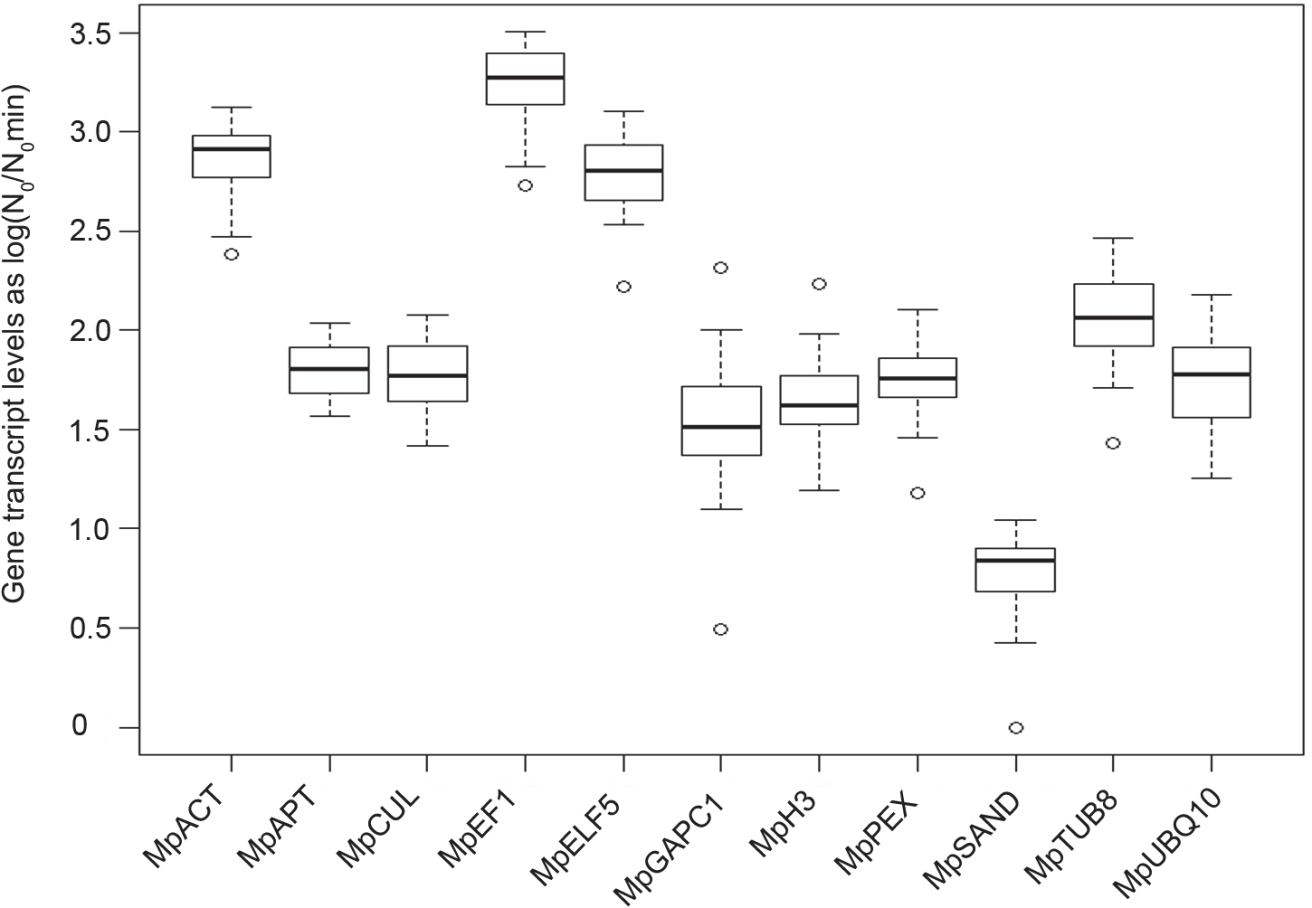
Range of transcript levels detected for each of the eleven candidate genes across all 22 conditions. Average N_0_ values per gene and condition (n = 3) were normalized on the smallest value then log transformed. Data were then represented for each gene as a box-plot.

To determine transcript levels for each candidate reference gene in each of the 22 biological contexts, LinRegPCR software [32] was used to compute mean efficiencies (E_m_) and initial cDNA amount (N_0_) values for every reaction. All E_m_ values were above 1.85 (**Table 1**), a value representative of an efficient amplification reaction [37]. **Fig. 1** shows a box plot of the log transformed N_0_/N_0min_ ratio calculated per gene across all conditions. The data reveal that *MpEF1α* transcript levels were the highest over all conditions tested whereas *MpSAND* levels were the lowest. Furthermore, *MpPEX* and *MpAPT* transcript levels varied least across the whole experiment whereas *MpGAPC1* and *MpUBQ10* transcript levels varied greatly depending on biological context. Importantly, however, transcript levels were not normalized by known reference genes and thus values are subject to biological and technical perturbations. Therefore, whilst this preliminary analysis showed that the amplification procedure was successful, further refinement was needed to identify reference genes.

### Assessment of variation in transcript levels for each candidate reference gene

**Fig 2:**
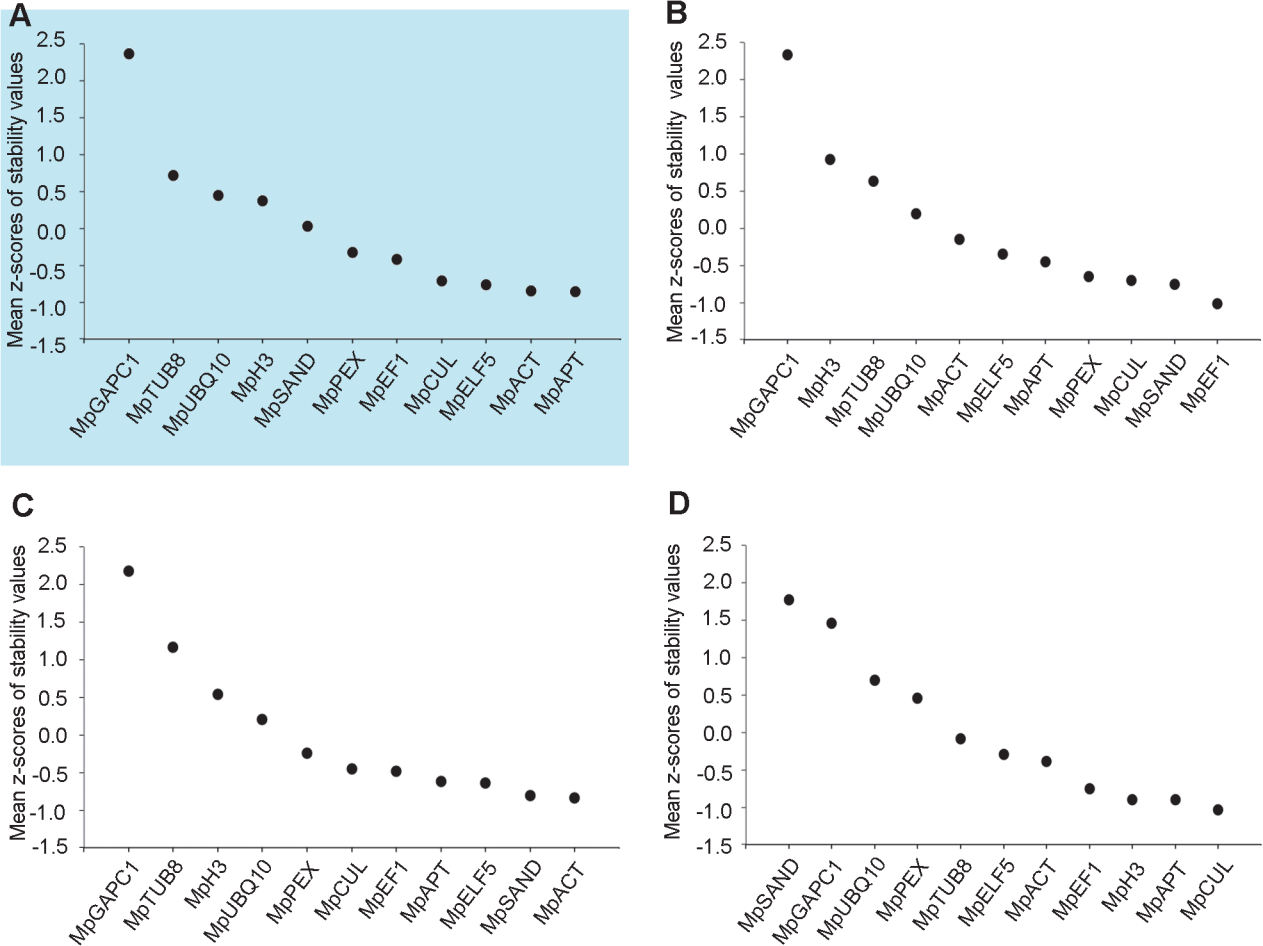
Average variation in transcript levels for each candidate gene. All 22 conditions tested (**A**, highlighted in blue), development group (**B**), abiotic stress group (**C**), and hormone group (**D**). Variation is represented as the average of z-scores derived from geNorm and NormFinder results. The lower the z-score, the more invariant the transcript levels between samples.

To assess variation in transcript levels across samples, qPCR data were analyzed in groups using geNorm [16] and NormFinder [15] algorithms. Groups were defined as ‘all’, ‘development’, ‘abiotic stress’ and ‘hormone’, which encompassed all biological contexts, developmental stages and organs, nutrient starvation and cold exposure, and hormone treatments respectively. Both approaches produced a ranked list of genes with the lowest values representing the most consistent transcript levels. Both geNorm and NormFinder ranked similar genes in the top five but not necessarily in the same order (**S2** and **S3 Tables**) (see for example *MpACT*, *MpAPT*, *MpCUL* and *MpEF1* when considering all biological contexts). However, in some cases important discrepancies existed between rankings produced by the two algorithms: for example *MpELF5* is ranked 1^st^ by geNorm but 7^th^ by NormFinder for the abiotic stress group. In an attempt to reconcile the discrepancies observed, we averaged the values obtained from the two algorithms. Values could not be compared directly as geNorm and NormFinder do not rely on the same calculations. Consequently, we averaged z-scores derived from the data produced by each algorithm for each gene, and for each group (**Fig. 2**). As for rankings produced by the two algorithms, the lower the z-score for a gene, the more consistent transcript levels are across samples.

When using z-scores, a different gene ranking was revealed for each group. Transcript levels of *MpGAPC1*, *MpUBQ10* and *MpTUB8* varied most across the four groups (**Fig. 2**). As such, these three genes are unsuitable for use as reference genes in qPCR assays. *MpEF1α* and *MpSAND* appeared to be suitable for developmental comparisons (**Fig. 2B**), *MpACT* and *MpSAND* for abiotic stress (**Fig. 2C**), and *MpCUL* and *MpAPT* for hormone treatments (**Fig. 2D**). Taking into account all biological contexts, *MpAPT* and *MpACT* had the lowest z-scores. In addition, *MpAPT* was ranked third by geNorm and second by NormFinder, and *MpACT* was ranked second and fifth—in this last case with a value almost identical to the value of the genes ranked third and fourth. Consequently, *MpAPT* and *MpACT* are the most suitable reference genes for generic studies. Notably, some genes appeared to have relatively constant transcript levels in some contexts but very variable levels in others. For example, *MpSAND* had a very low z-score when assayed in the development and abiotic stress groups (**Fig. 2B** and **C**) but the highest score in the hormone group (**Fig. 2D**). In contrast, *MpH3* had a low score in the hormone group but a very high score in all other cases.

### Estimation of the number of reference genes required for normalization

**Fig 3:**
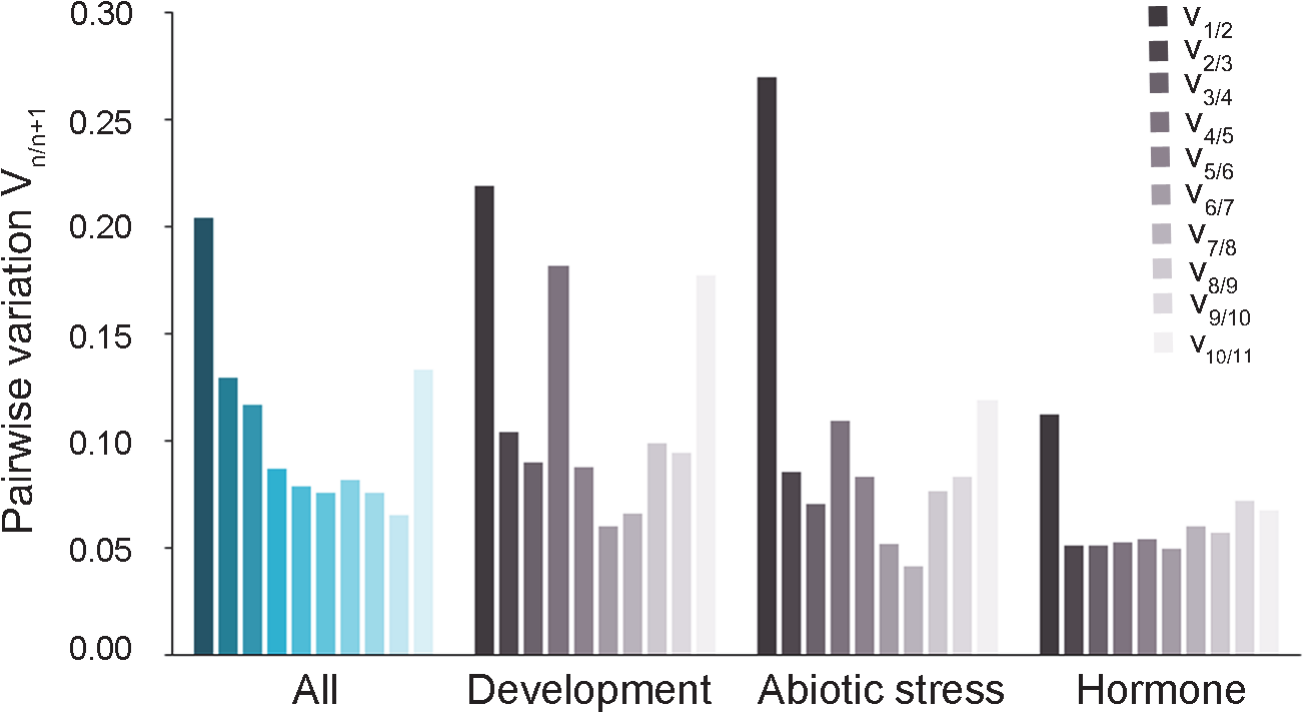
Estimation of the optimal number of reference genes required for accurate qPCR data normalization. Estimation was done by calculating the pairwise variation (V_n/n+1_) of the normalization factors NF_n_ and NF_n+1_ as described in Vandesomple et al., [16].

To further improve normalization strategies, several reference genes can be used simultaneously. Vandesompele *et al*. [16] described a method to estimate the optimum number of genes needed that computes the variation V_n/n+1_ induced by the inclusion of the (n+1)^th^ best ranked gene in the calculation of the normalization factor. A variation greater than 0.15 was assumed to be significant [16]. We implemented the method as a Perl script (available as a supplemental file) and, for each group of biological contexts, computed the stepwise variation obtained when a new gene was added to the normalization factor, starting from the best to the worse ranked gene. When adding the second ranked gene to the normalization factor (V_1/2_), a variation greater than 0.15 was obtained for every group except the hormone group (**Fig. 3**). Using the third ranked gene in addition to the first two decreased the variation again but did not improve any of the normalization factors calculated (V_2/3_ < 0.15 in all cases). Adding more genes tend to make the stepwise variation rise again for three of the four groups, an observation previously reported (see for example Vandesompele *et al*. [16]). Since the variation when adding the third best-ranked gene was below 0.15, we concluded that using the two most highly ranked genes to compute the normalization factor was sufficient.

### Validation of reference genes for qPCR analysis during nitrate and phosphate starvation

**Fig 4:**
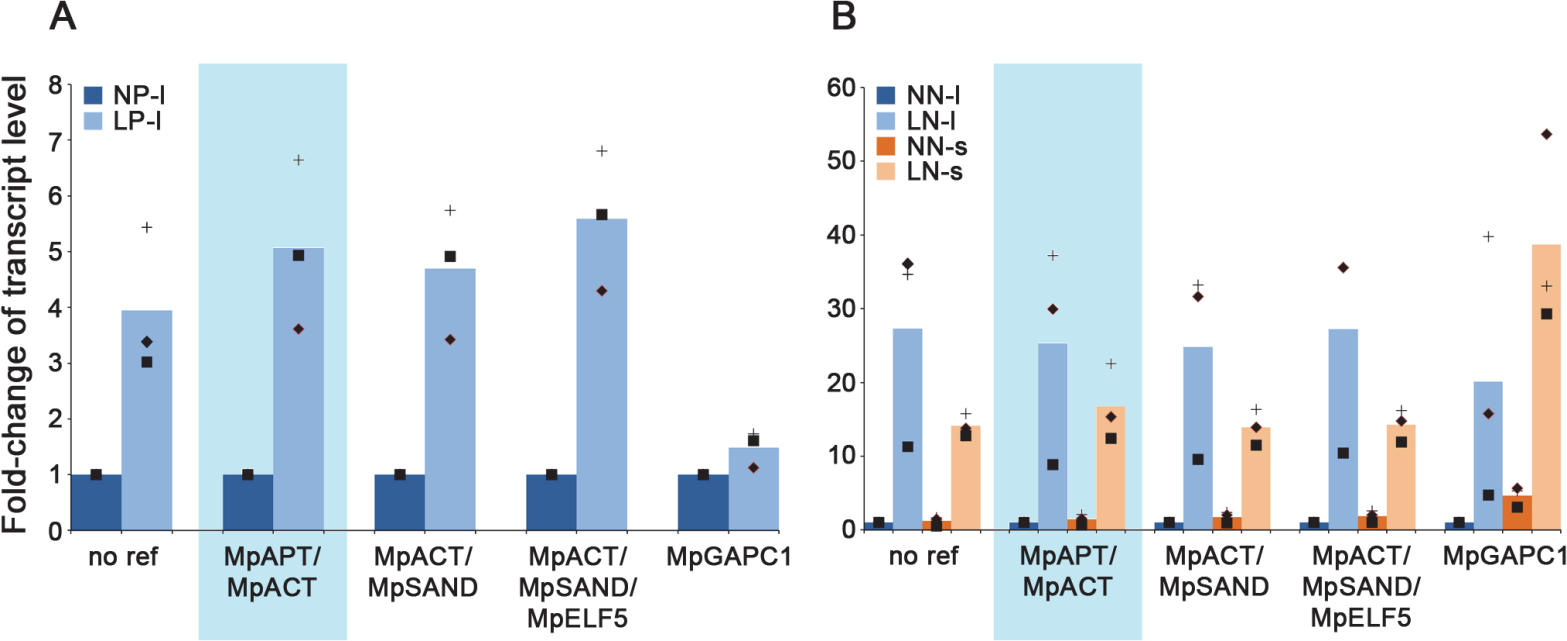
Transcript levels of MpPHT1-1 and MpNRT2-1 after phosphate and nitrogen starvation. Histograms represent the fold-change of the average transcript levels (n = 3) compared to the long-term control condition of *MpPHT1-1* (**A**) and *MpNRT2-1* (**B**). Data were non-normalized (‘no ref’) or normalized by a factor consisting of the geometric mean of the transcript level of *MpAPT* and *MpACT* (highlighted in blue), of *MpACT* and *MpSAND*, of *MpACT*, *MpSAND* and *MpELF5*, and only by the transcript level of *MpGAPC1*. Biological replicates are depicted superimposed with crosses, diamonds and squares, respectively for replicate one, two and three. NP stands for normal phosphate, LP for low phosphate, NN for normal nitrate, and LN for low nitrate media respectively. A ‘s’ indicates a short (24 h) treatment, a ‘l’ a long (17 days) treatment.

In *Arabidopsis*, *AtPHT1-1* (*PHOSPHATE TRANSPORTER* 1) that encodes a phosphate transporter and *AtNRT2-1* (*NITRATE TRANSPORTER 2*) that encodes a nitrate transporter involved in the High Affinity Transport System are induced during phosphate and nitrate starvation, respectively [38,39]. We identified a similar sequence for each gene in our *M*. *polymorpha* transcriptome dataset and measured transcript levels after nutrient starvation (**Fig. 4**). Non-normalized qPCR data showed a ∼4-fold increase in *MpPHT1* transcript levels after phosphate starvation, and a ∼27-fold or ∼14-fold increase in *MpNRT2* transcript levels after long term or short term nitrate starvation (**Fig. 4A** and **B**, ‘no ref’). The induction of both genes transcription is consistent with previously published results in *Arabidopsis* [38,39]. Given the dramatic differences detected in non-normalized qPCR between transcript levels before and after starvation, these two genes were deemed appropriate for validation of our candidate reference genes in *M*. *polymorpha*.

**Fig. 4** illustrates different strategies of normalization. Using *MpAPT* and *MpACT*, which we showed to be the best candidate reference genes for generic studies, *MpPHT1* and *MpNRT2* transcript levels exhibited a profile reminiscent of non-normalized data, with fold-change values in the same range: ∼5-fold increase for *MpPHT1*, and ∼25-fold or ∼17-fold increase (long or short term starvation) for *MpNRT2* (**Fig. 4A** and **B**, highlighted in blue). The same profile was observed when the data were normalized with the two best candidate genes for the abiotic stress group (*MpACT* and *MpSAND*), with a small change, if any, in the fold-change increase (∼5-fold, ∼25-fold and ∼14-fold). Addition of the third ranked gene of the abiotic stress group (*MpELF5*) to normalize the data only marginally changed the obtained fold-change increase for both *MpPHT1* and *MpNRT2* after starvation (**Fig. 4A** and **B**). This result confirmed that two reference genes are sufficient to normalize qPCR data in *M*. *polymorpha*. In combination, these data demonstrate that the two generic reference genes, as well as the two genes specific to the abiotic stress group can be used to reliably normalize qPCR data across abiotic stress contexts.

Data normalization was also carried out using the most variable gene, *MpGAPC1*. Drastically different (and obviously inaccurate) transcript levels were obtained for both *MpPHT1* and *MpNRT2*. For example, *MpPHT1* transcript levels appeared to be equivalent in high and low phosphate contexts, as opposed to being higher in low phosphate. Similarly, whereas in our experiment *MpNRT2* transcript levels are higher after long-term nitrate starvation than after 24 hours (non-normalized data and data normalized with the best candidate reference genes), normalization with *MpGAPC1* reversed the trend.

These results demonstrate that the methods used here for the identification of reference genes are robust, and also that improper normalization can lead to erroneous analysis of qPCR raw data.

## Discussion

Normalization of qPCR results is needed to correct for biological and experimentally introduced variability. A common method of normalization is to compare transcript levels of the gene of interest to those of a reference gene that, through either transcriptional or post-transcriptional mechanisms, is characterized by relatively constant transcript levels across the conditions and/or tissue types being analyzed. Selection of suitable reference genes is not a trivial task and requires careful analysis of non-normalized transcript levels. To facilitate this task, several algorithms such as geNorm and NormFinder are available [15,16]. Here we applied both algorithms, and averaged their results, to identify a set of suitable reference genes for qPCR studies in the model liverwort species *M*. *polymorpha*.

We tested eleven candidate genes in a broad range of experimental conditions. We show that genes such as *GAPC1*, *TUB*, or *UBQ* that are commonly used in other organisms [4,5,20] are poor reference genes in *M*. *polymorpha*. Instead, our results indicate that for generic applications, normalization using *MpAPT* and *MpACT* is optimal. Orthologs of both genes have been shown to be good reference genes in previous studies with other species [4,5,23,25].

The suitability of any particular gene as a reference for normalization can be surprisingly variable depending on biological context. For example, *MpSAND* transcript levels are elevated after hormone treatments, rendering the gene unsuitable for normalization in these conditions. However, *MpSAND* transcript levels are relatively constant under different growth conditions, after nutrient starvation and cold response and in different developmental contexts. This constancy has also been observed in other organisms such as *Eucalyptus* or *Arabidopsis* [20,25]. This example demonstrates that where possible, reference genes should be determined for each subset of conditions, as restricted as possible; a conclusion also drawn previously by others [5,18].

An important question that arises when testing for reference genes is the number of genes necessary for optimal normalization. The authors of geNorm recommend the use of at least three reference genes (and up to five in some cases) in order to compensate for individual reference gene variation in different study conditions [16]. In contrast, NormFinder authors argue that the use of several reference genes does not necessarily improve normalization accuracy [15]. The use of multiple reference genes increases the number of qPCR reactions required for any particular experiment. This can become problematic for large-scale projects or when working with material from which only small amounts of RNA can be extracted. Here we have shown that two reference genes are sufficient to produce a reliable normalization factor.

## Conclusion

We report the identification of a reference gene set suitable for normalizing transcript level data obtained by qPCR in *Marchantia polymorpha*. We showed that two reference genes were necessary and sufficient to normalize qPCR data and determined the best gene pairs for different experimental conditions such as hormone treatments and abiotic stress, and across a variety of developmental stages. While different pairs were found to be the most appropriate for these biological contexts, we observed that the *MpAPT* and *MpACT* combination was the best for generic studies. Therefore, we recommend the use of *MpAPT* and *MpACT* for *Marchantia polymorpha* qPCR analysis.

## Supporting Information

S1 Fig Phenotype of exogenously treated plants. 17 days mock (A) and 1 μM ABA (B) treatments, mock (C) and 750 nm NAA (D) treatments, high (E) and low (F) phosphate, high (G) and low (H) nitrate. Scale bars equals to 1 cm (A, B, G, H) or 2 cm (C, D, E, F).
(TIF)

S2 Fig Typical dissociation curves for each amplicon. Pictures were taken using the qPCR instrument’s software: *MpAPT* (**A**), *MpACT* (**B**), *MpPEX* (**C**), *MpUBQ10* (**D**), *MpCUL* (**E**), *MpELF5* (**F**), *MpEF1α* (**G**), *MpTUB8* (**H**), *MpH3* (**I**), *MpGAPC1* (**J**), *MpSAND* (**K**), *MpPHT1* (**L**), *MpNRT2* (**M**).
(TIF)

S1 Table RNA integrity number obtained for the three biological replicates of each sample. (DOCX)

S2 Table Transcript level stability values calculated by geNorm algorithms for each reference gene. (DOCX)

S3 Table Transcript level stability values calculated by NormFinder algorithms for each reference gene. (DOCX)

S1 Data N_0_ data in excel file. (XLSX)

S2 Data *M*. *polymorpha* gene sequences in word file. (DOCX)

S3 Data Perl script implementing V_n/n+1_ calculations. (TXT)
