# Supplementary figures and images for: Identification of Reference Genes for Real-Time Quantitative PCR Experiments in the Liverwort Marchantia polymorpha
Source: PLoS One. 2015 Mar 23;10(3):e0118678. doi: 10.1371/journal.pone.0118678 (PMC4370483; doi:10.1371/journal.pone.0118678)

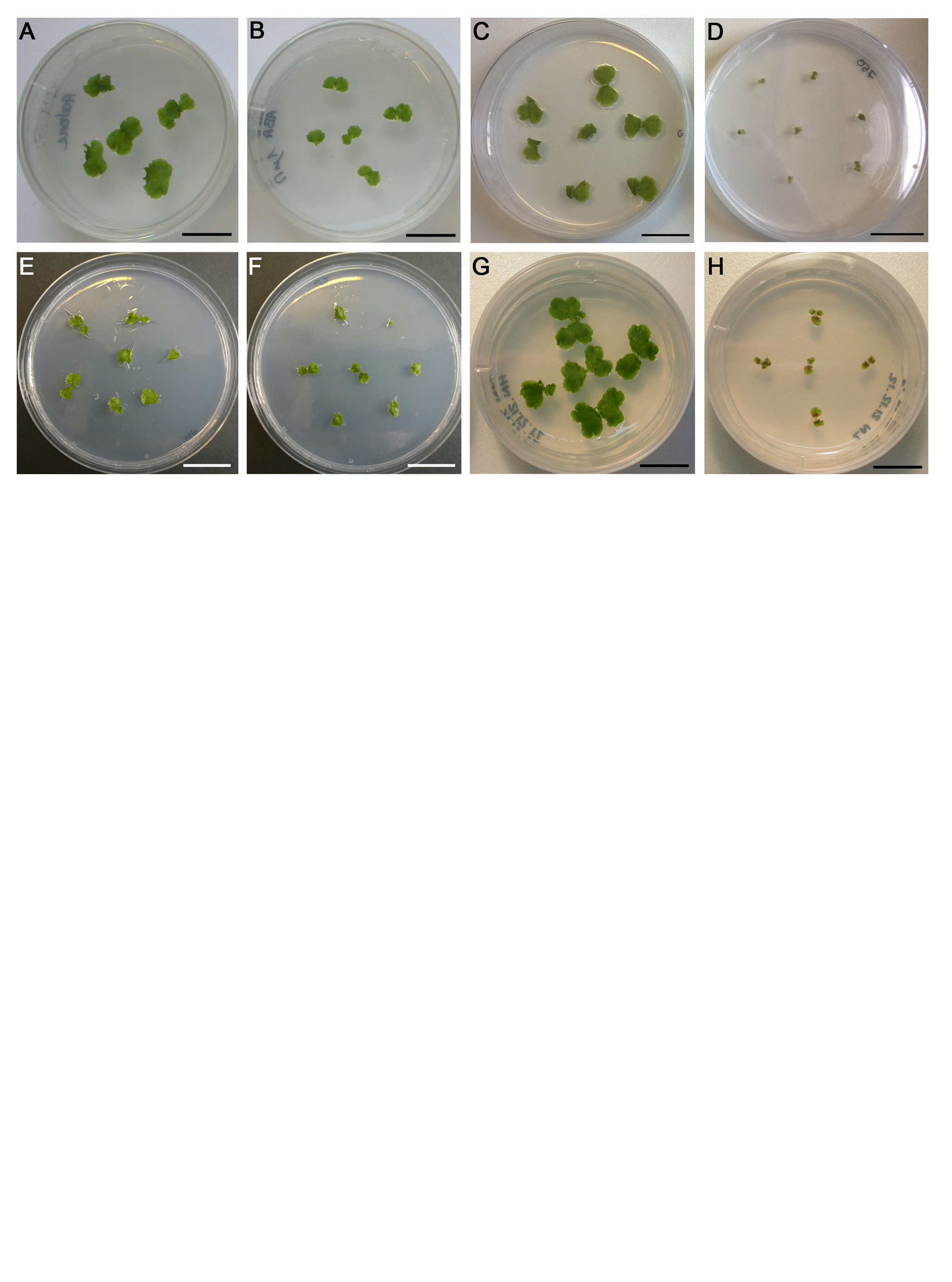

Supplement: S1 Fig — 17 days mock (A) and 1 μM ABA (B) treatments, mock (C) and 750 nm NAA (D) treatments, high (E) and low (F) phosphate, high (G) and low (H) nitrate. Scale bars equals to 1 cm (A, B, G, H) or 2 cm (C, D, E, F). (TIF) [file pone.0118678.s001.tif]

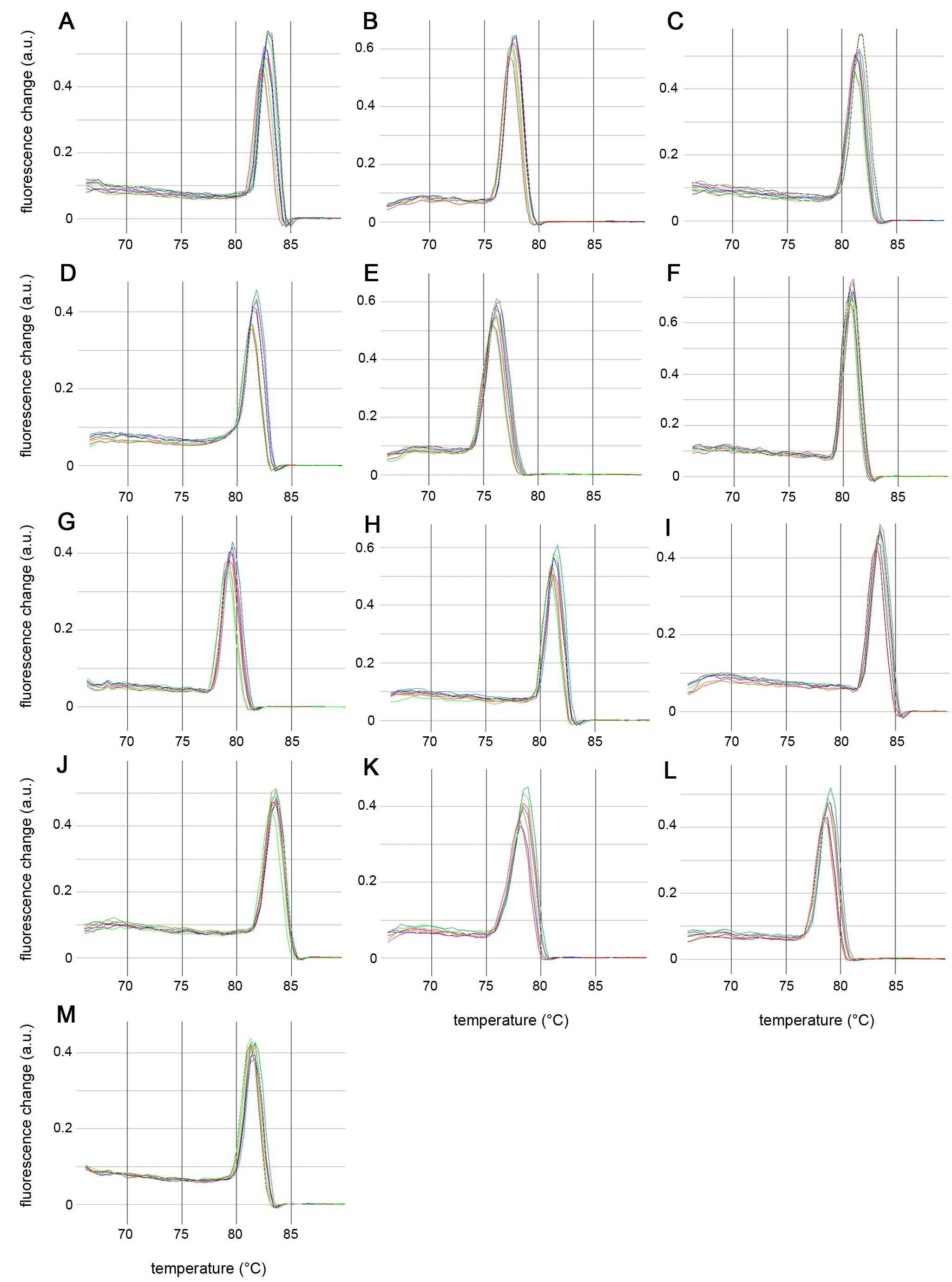

Supplement: S2 Fig — Pictures were taken using the qPCR instrument’s software: MpAPT (A), MpACT (B), MpPEX (C), MpUBQ10 (D), MpCUL (E), MpELF5 (F), MpEF1α (G), MpTUB8 (H), MpH3 (I), MpGAPC1 (J), MpSAND (K), MpPHT1 (L), MpNRT2 (M). (TIF) [file pone.0118678.s002.tif]
