# Supplementary material for: Identification of Reference Genes for Real-Time Quantitative PCR Experiments in the Liverwort Marchantia polymorpha
Source: PLoS One. 2015 Mar 23;10(3):e0118678. doi: 10.1371/journal.pone.0118678 (PMC4370483; doi:10.1371/journal.pone.0118678)
Supplement: S1 Table — (DOCX) [file pone.0118678.s003.docx]

**Table S1. RNA integrity number obtained for the three biological replicates of each sample.**

| **Group** | **Sample** | **Replicate 1** | **Replicate 2** | **Replicate 3** |
| --- | --- | --- | --- | --- |
| ***Development*** |  |  |  |  |
|  | WP G 17d | 8.3 | 7.7 | 8.6 |
|  | WP G 24d | 8 | 8.4 | 8.7 |
|  | WP soil 17d | 7.6 | 8.5 | 8.8 |
|  | WP soil 24d | 8.4 | 8.7 | 8.9 |
|  | Gemmae | 8 | 9 | 9.2 |
|  | Antheridiophore | 9 | 9.5 | 9.4 |
|  | Archegoniophore | 8.1  1 | 9 | 8.8 |
| ***Abiotic stress*** |  |  |  |  |
|  | WP HP | 8.8 | 8.8 | 8.9 |
|  | WP LP | 8.7 | 8.9 | 8.8 |
|  | WP HN | 8.5 | 8.8 | 8.6 |
|  | WP LN | 7.9 | 9.1 | 9 |
|  | WP HN +24h HN | 8.8 | 8.7 | 8.9 |
|  | WP HN +24h LN | 9 | 8.7 | 9 |
|  | WP G +24h cold | 7.5 | 8.5 | 8.4 |
| ***Hormone*** |  |  |  |  |
|  | WP G mock | 9 | 8.9 | 8.8 |
|  | WP ABA 1µM | 8.8 | 8.9 | 8.9 |
|  | WP G mock | 7.2 | 8.5 | 8.5 |
|  | WP NAA 750 nM | 7.8 | 8.5 | 8.4 |
|  | WP +24h mock | 8.8 | 8.7 | 8.9 |
|  | WP +24h ABA 2µM | 8.8 | 8.7 | 8.7 |
|  | WP +24h NAA 2µM | 9 | 8.8 | 8.7 |
|  | WP +24h GR24 1µM | 8.9 | 9 | 8.9 |
