# Supplementary material for: Identification of Reference Genes for Real-Time Quantitative PCR Experiments in the Liverwort Marchantia polymorpha
Source: PLoS One. 2015 Mar 23;10(3):e0118678. doi: 10.1371/journal.pone.0118678 (PMC4370483; doi:10.1371/journal.pone.0118678)
Supplement: S2 Table — (DOCX) [file pone.0118678.s004.docx]

**Table S2: Transcript level stability values calculated by GeNorm algorithms for each reference gene.**

|  | **All** | | **Development** | | **Abiotic stress** | | **Hormone** | |
| --- | --- | --- | --- | --- | --- | --- | --- | --- |
|  | Gene | Stability | Gene | Stability | Gene | Stability | Gene | Stability |
| **1** | *MpELF5* | 0.3775 | *MpSAND* | 0.3490 | *MpELF5* | 0.2657 | *MpH3* | 0.1526 |
| **2** | *MpACT* | 0.3775 | *MpEF1* | 0.3490 | *MpACT* | 0.2657 | *MpAPT* | 0.1526 |
| **3** | *MpAPT* | 0.4253 | *MpPEX* | 0.3815 | *MpEF1* | 0.3270 | *MpCUL* | 0.1985 |
| **4** | *MpCUL* | 0.4741 | *MpCUL* | 0.4064 | *MpSAND* | 0.3489 | *MpEF1* | 0.2151 |
| **5** | *MpEF1* | 0.4926 | *MpAPT* | 0.4994 | *MpAPT* | 0.3962 | *MpACT* | 0.2280 |
| **6** | *MpPEX* | 0.5123 | *MpELF5* | 0.5216 | *MpCUL* | 0.4206 | *MpTUB8* | 0.2780 |
| **7** | *MpSAND* | 0.5382 | *MpACT* | 0.5653 | *MpPEX* | 0.4417 | *MpELF5* | 0.3068 |
| **8** | *MpH3* | 0.5852 | *MpUBQ10* | 0.6215 | *MpUBQ10* | 0.4852 | *MpUBQ10* | 0.3547 |
| **9** | *MpUBQ10* | 0.6249 | *MpTUB8* | 0.6970 | *MpH3* | 0.5394 | *MpPEX* | 0.3951 |
| **10** | *MpTUB8* | 0.6546 | *MpH3* | 0.7623 | *MpTUB8* | 0.6064 | *MpGAPC1* | 0.4628 |
| **11** | *MpGAPC1* | 0.7923 | *MpGAPC1* | 0.9541 | *MpGAPC1* | 0.7266 | *MpSAND* | 0.5188 |
