# Supplementary material for: Identification of Reference Genes for Real-Time Quantitative PCR Experiments in the Liverwort Marchantia polymorpha
Source: PLoS One. 2015 Mar 23;10(3):e0118678. doi: 10.1371/journal.pone.0118678 (PMC4370483; doi:10.1371/journal.pone.0118678)
Supplement: S3 Table — (DOCX) [file pone.0118678.s005.docx]

**Table S3: Transcript level stability values calculated by NormFinder algorithms for each reference gene.**

|  | **All** | | **Development** | | **Abiotic stress** | | **Hormone** | |
| --- | --- | --- | --- | --- | --- | --- | --- | --- |
|  | Gene | Stability | Gene | Stability | Gene | Stability | Gene | Stability |
| **1** | *MpCUL* | 0.2142 | *MpEF1* | 0.1745 | *MpSAND* | 0.1605 | *MpCUL* | 0.0903 |
| **2** | *MpAPT* | 0.2489 | *MpCUL* | 0.3460 | *MpAPT* | 0.1710 | *MpEF1* | 0.1818 |
| **3** | *MpEF1* | 0.3686 | *MpAPT* | 0.3496 | *MpCUL* | 0.2306 | *MpELF5* | 0.2208 |
| **4** | *MpPEX* | 0.3748 | *MpELF5* | 0.3951 | *MpPEX* | 0.3232 | *MpAPT* | 0.2281 |
| **5** | *MpACT* | 0.3846 | *MpSAND* | 0.4367 | *MpACT* | 0.3355 | *MpH3* | 0.2317 |
| **6** | *MpELF5* | 0.4447 | *MpPEX* | 0.4551 | *MpEF1* | 0.4293 | *MpACT* | 0.2896 |
| **7** | *MpSAND* | 0.5521 | *MpACT* | 0.4767 | *MpELF5* | 0.4710 | *MpTUB8* | 0.3605 |
| **8** | *MpUBQ10* | 0.6024 | *MpUBQ10* | 0.6795 | *MpUBQ10* | 0.5254 | *MpPEX* | 0.3891 |
| **9** | *MpH3* | 0.6557 | *MpTUB8* | 0.9185 | *MpH3* | 0.6216 | *MpUBQ10* | 0.5604 |
| **10** | *MpTUB8* | 0.7035 | *MpH3* | 1.0462 | *MpTUB8* | 0.8926 | *MpGAPC1* | 0.6994 |
| **11** | *MpGAPC1* | 1.4564 | *MpGAPC1* | 1.9426 | *MpGAPC1* | 1.3029 | *MpSAND* | 0.7367 |
