# Supplementary material for: Identification of Reference Genes for Real-Time Quantitative PCR Experiments in the Liverwort Marchantia polymorpha
Source: PLoS One. 2015 Mar 23;10(3):e0118678. doi: 10.1371/journal.pone.0118678 (PMC4370483; doi:10.1371/journal.pone.0118678)
Supplement: S2 Data — (DOCX) [file pone.0118678.s007.docx]

**Data S2**

>MpSAND

GGAGCCGTGAATCCTCCACGTCGAAGGACGAGGAGCGACGAAATCTGCAGTGCGGCTGCAACTGCAGGCGATGGAAAGGAGCAGGCGGAGGTCGAGCTCCCTTTGAATCATTTCCCGTTCATGAAGTTTCGTTTTTCGTCTCATATCATCTCGTCTCGTCTTTCTGTTTGTCTGTCTTTCTGTCTGGTTCCTGTTGATGTCTGACTCGGGGCTCGCCTGAAAGAGAGGCCGCGAGCTGCTGATCGGCAGACGATGCGGAGGATGCGGTGCCCGGGGCTTTCATTCGATCCATCTTCGCTGCAGCTGCACTGCTGCTGCTGCTCCGAGAAGAGCATCGACTGGAGAGAAGAAGAAGAGATTATTCGATGAGTTCTCCTCTTGATGAGCTTCGGAGGACAGCGTGGTTCGTGAATCCGGCTGTTCGTTCTCGAGAGGTCCGATCCAAGAATTGACTGATTCCACTCCCCCCTGCCCCCTGCGACGTCTCTTGCACGATGGGTCGGTTTACCTCGGGCGATCTGTGCATGGGGGACACTGGCCACTGGTCCCTCTGTCGGATGAGCTCGGGTGGATCATCGGACTACTCCTGTTTGTCGGAAGCCTGATCTTTTCTCTCGATTTTTCTCTTCTCCATTTCGTTAGGGTGAGGGCTACAAGGACGTGCCCGACTTTCAATTTGAGCTCATCTTTTGCCGTAGGCTTTGGGTTTCAGTGGCGATTCTTATCAGGAAAAGCTCTGCTCACCGGCTCGTCGTCGCAGCTGGCATCTCGTGCGTCTGCCCTTGTGTAGCTGGCGGGTAAGCTCTTGGCTCAATGA**ATG**GCATCATCAGGTGGTAAGGATTCTTCGAGCCCGATAGATTCGAATGGTAGGGTTGGAAGGGAGCATTCAAATCCGGAGGCGATATCGGCATTGTACGAGGACCTTTTTGAGAACGATAATAGTGAGCGTCCGTGCTACTTGCCAGACTCAGGGCAAGAGGATTACAGCGGCGATGGAGATGGAGTTGGAGATGGAGATGAGGATGGAAACAGTCCCCGTGAGATTGTCCAGAAGGGAAGAGGAGACAGCGTGGTTGAGGAGAAAGGGGAATTGAATGCGGCGAACGGAGCTTCACCCGAAGCGTCAGCCACTCAGATGAACAATGATAGCAGGGAAAGCGGGTCGGATGTCGGGACTTTGCCGGAGGATCTCGACGCAAG

GTCCAGTACAGGGAACGAAGACGAAATACAGGAAAAAGATCATGAGTTTAACTTCAGAGATAGAGTTCGTGCGGAATCTTCATCAACGGAACGCTTGGTACATTTAGATGGAGAAATGTCTTCGGATCGTGGTTTAGATGGTCCCCCCAGTCCAACCAGCAGCCAAGGGGGGACTAGCGGCTATATGGCAGGAAGTGGTAGTAGTAGTGCGAGTACGAGCAGCGTCAGTGGTGTAGAAATCGGTACTGATCAAGATACTGAAGGAATTGGAAGCAGGAAGGAGGAGCGGGGAGTATGGGTTCCAGGAAAACGTCATTCTCAGGAGGATGATAGTCATCCTTCCTGGCGACGTCACAAGAAGCACTTTTTCATACTCAGTAATGCAGGAAAACCAATCTATTCCAGATATGGGGATGAACATAAACTTGCAGGGTTTTCTGCGACCCTTCAGGCCATAATGTCATTCGTAGAAAACAGTGGGGATACGATACACCTTGTCCGGGCAGGAGATCATCAGATAATCTTTTTGGTTAGAGGTCCATTGTATCTGGTCTGTATCAGTTCCACTGATGAACCTTTCCAAGTCCTCAAAGGCCAAAAAAAAAAACTGGTCTGTATCAGTTCCACTGATGAACCTTTCCAAGTCCTCAAAGGCCAGTTAGAGCTTCTGCATGGACAGCTGCTATTGATCTTAACGAAGGCAGTCGAAAAGTGCTTCTTGAAGAACTCAAAATTTGACATGAGACCGCTCCTTGGAGGCACGGATGTTGTTTTCTCCTCGTTGATTCATGCCTTCAGCTGGGATCCAGCAACCTTTTTGAGTGCCTATTCGTGCCTACCTCTTCCCTACGTCACAAGACAGGCTGCTTGTGGTGCTCTTCAGGACATGACGGACTCCGGAGTTCTGTTCGCTTTGTTGATGTGTGGCACAAAGGTGATAAGTCTCGTCGGTCCGCGGAAAGCTTCACTTCATCCCCATGATCTGTTGCTGTTATCCAATTTTATCTTGTCTTCTGATTCATTCAGAACATCCGAATCATTTTCTCCCGTATGCCTGCCTCGATACAATGCCACAGCATTTATGTATGCCTATGTGCAGTACCTCAATAAAGACACTTGTTTGATCTTACTTACTGGGGACCCCAACGACTTTTTCCACTTAAAAGAATGCAGAGCACAAATTGAGATTGCTTTGAGGGA

GTCAGATGTTTTGCGGGAGGTGAACAATTCAATATCACGAGGTGGATTGAGAATAGAGGACTTGCCGGATTCAGAGGACCTAGTACCCCCTGAAGGTGTATCCAGTAGGGAAGACAGATCTAGTGGTGATGGAGCTAGCACATCTGCATTTAACTCTGTTCCCGGAGCCGGCGGAGCTGCAGGCCTTTGGCATTTTATTTATCGCAGCACATACTTGGACCAGTTTGTGGCCTCGGAATTTTCACCCCCTTTGAATACACGCTCGGCTCAAAAGAGACTATACAGAGCTTTCCAAAAGCTGCATGCTTCAATGCATGATACCGAAGCATCAGGACCTCACAAGATGCAATATAGGAGAGATGAAAATCATGTTCTGCTTTCGTGGAGAAACTCCGATTTCGAGCTCTACGCTGCATTTGATCCTCTCGCCGAGAAAAGCTCTGCAATATTTGTATGCAACCGAATTTGCCAATGGATTCGAGATGTGGAATGCGAAATATTTTTATTAGGTGCAACACCATTTAACTGG**TGA**GTTCCATGCAACACAAACGGCCTTGTAAACTCATACGGTATCTGTTTTACGGATATATATCTCAGAATGCATGGGACAACGTGATTCTTTTGTAAAATTCTACCCGGTCTTGGCCCGGTAGCTGGTTTTACGCATCAACCATATGAATACTCTCAATGGTTGCAACTTTCCACTGCCAA

>MpEF1a

ATGACATATGAACTGAAGATCTGCAGAAAAGCAGGATGCTGCAGAATCTCAGTCGGGAGGTGTGGTTCGATCCCATCGGGTGTAGTTGAATTAGATTTACCGTAGAGGACAAGGTTTGTGGCTCTCGGTCCATTCGCTTAAGATGTAGCTCGGACGTTTTCCAGGCCCATGACATCTTCTCTCTCTCCGAATTCGCTCGCAGTCGATCGATCGTATCTGTCTTCCGGCTCCTGCTTTCGGTAATCGAGCAACATGGGTAAGGAGAAGTTTCATATCAACATCGTGGTTATTGGCCATGTCGACTCCGGGAAGTCGACCACCACTGGTCACCTTATTTACAAGCTGGGAGGTATCGACAAGCGTGTCATCGAGCGGTTTGAGAAGGAAGCTGCTGAAATGAACAAGCGTTCTTTCAAGTACGCTTGGGTGCTTGACAAGTTGAAGGCTGAGCGTGAGCGTGGTATTACTATCGATATCGCTCTCTGGAAGTTCGAGACCAACAAGTATTATTGCACAGTCATCGATGCCCCCGGTCACAGGGATTTCATCAAGAATATGATTACTGGAACTTCCCAGGCTGATTGCGCCGTTCTCATCATCGACTCAACCACCGGAGGATTCGAGGCAGGTATCTCTAAGGATGGCCAGACCCGAGAGCACGCTCTCCTGGCTTTCACTCTGGGTGTGAAGCAGATGATCTGCTGCTGCAACAAGATGGATGCAACCACCCCGAAGTACTCGAAGGGCCGTTACGACGAGATCATCAAGGAGGTGGCTAACTATTTGAAGAAGGTCGGGTACAACCCCGACAAGATTCCCTTCGTGCCAATTTCTGGATTCGAAGGAGACAATATGATTGAGCGATCTACTAACCTTGACTGGTACAAGGGACCTACCCTCCTCGAAGCTTTGGACAGCATCAGTGAGCCTAAGAGGCCTTCCGACAAGCCCCTCAGGTTGCCCCTTCAGGATGTGTACAAGATTGGAGGAATTGGAACTGTCCCAGTGGGACGAGTGGAGACTGGTGTGATCAAGCCAGGTATGGTGGTGACGTTTGCTCCCACCGGTCTGACCACTGAGGTCAAGTCCGTCGAGATGCACCACGAAGCTTTACTCGAGGCTCTGCCCGGTGACAACGTCGGATTCAATGTGAAGAATGTCGCTGTCAAGGACTTGAAGAGAGGATTTGTTGCATCCGAC

TCCAAGAACGACCCTGCCAAGGAGGCTGCGAACTTCACCTCCCAGGTTATCATCATGAACCACCCGGGACAGATCGGAAACGGATACGCCCCAGTTCTCGACTGCCACACTTCCCACATTGCAGTGAAGTTTTCCGAGATCCTGACCAAGGTCGATCGTCGATCAGGAAAGGAAATTGAGAAGGAACCAAAGTTTTTGAAGAATGGAGACGCCGGATTCGTTAAGATGATTCCCACCAAGCCCATGACTGTGGAGACCTTCGCTGAGTACCCACCTCTTGGACGATTCGCCGTGCGCGACATGAGGCAGACCGTCGCCGTCGGAGTTATCAAAGCCGTCGAAAAGAAGGAGCCCTCTGGCGCCAAGGTCACCAAGGCCGCCGCCAAGAAAAAGTGAATCGGGGCGACTGACATCACATTCCGGATAACGGACGATCCTGAATCTGTTGACCGTTACGACAGATGCGCTAAGCCTTGCCGATTGCTCGATAGGAGGAGGTTTCTTGCCCGCCTTCAGCAGACTTGGGTCCTAGACAGGCGGTGGCAAGGCTTGGAGGACACCGTAGGATATGTTTCGTGATTATGGTGTTAAAGTCGATTTTGACACATTGAGTTCTGTATCATTTTCATATCAACCTTTACCACTCTGTTTCGTATTCGTCCAAACACATCATATGTGTCCTGGTGAATGAGGACTATGGTTGAGCTGAGTGTATAATGAATAGCGAGTGTTCATGGTGTGGAATGTGATGTTGATATAGTGTACGCGTAGCTTTTAGAAACTTGTGTAGGAAGTAAATACCAGCATCTGTGCAATTTTATCATTCTACGCTCACTGCTGATGGAAATGTAATGAAAAAATTTGCACAACACTTGATACTCATTATTAGCGGTAAATTTGTCTTACAACTACAAAAACGAAGCGACACAAATAAGATAAGTACAGCAGTGAAGGAAAAATATTAATCATGAGTAAACAAGAAAAAGTAAAAAAGAAAAAAAAATCAATTTGTAAAATGAAATAAACTACCTATTATCACCACCATGTTTTCTACCCCAAAAATCAGAATGGGAAATTGCCTTA

>MpACT

ATGGCTGACTCTGAGGATGTGTCGCCTTTGGTGTGCGACAACGGATCTGGAATGGTCAAGGCTGGTTTCGCTGGAGACGATGCTCCCCGTGCAGTGTTCCCCAGTATTGTCGGAAGACCTCGCCACACCGGAGTCATGGTTGGTATGGGACAGAAGGATGCATATGTCGGAGATGAGGCCCAGTCCAAGCGTGGTATCCTGACTTTGAAGTATCCAATTGAACACGGTATCGTAACTAACTGGGATGATATGGAAAAGATCTGGCATCACACCTTCTACAACGAGTTGCGAGTTGCACCTGAAGAGCACCCGGTCCTTCTGACTGAAGCACCGTTGAACCCTAAGGCTAATCGTGAGAAGATGACCCAAATTATGTTCGAGACCTTTAACGTCCCCGCGATGTACGTGGCCATCCAGGCTGTGCTGTCTTTGTACGCCAGTGGTCGTACTACCGGTATTGTTTTGGATTCTGGTGATGGTGTCACTCACACCGTCCCAATTTACGAAGGTTACTCTCTTCCCCACGCCATCCTGAGGTTGGATCTTGCCGGACGTGATCTTACCGACGCTTTGATGAAGATCTTGACCGAGAGAGGTTACTCTTTCACCACCACCGCAGAGCGTGAAATTGTGAGAGACATGAAGGAAAAGCTTGCATATGTTGCACTTGACTTCGAGCAAGAGCTTGACACCTCCCGATCCAGCTCCTCACTGGAAAAGAGCTACGAGCTTCCCGACGGTCAAGTTATCACCATTGGTGCCGAAAGATTCAGGTGCCCAGAGGTTCTGTTCCAACCCTCATTGATCGGTATGGAGGCATCTGGTATCCACGAGACCACCTACAATTCAATCATGAAGTGTGACGTGGATATCCGTAAGGACTTGTATGGAAACATTGTGTTGTCTGGAGGAACGACCATGTTCCCAGGTATCGCCGAGCGTATGACCAGGGAAATCACCAGCCTTGCTCCCAGCAGCATGAAGATTAAGGTTGTTGCACCACCAGAGAGGAAGTACAGCGTCTGGATCGGAGGATCTATCTTGGCTTCTCTCAGCACCTTCCAGCAGATGTGGATCGCCAAGGCCGAGTACGATGAGTCGGGCCCATCTATCGTCCACAGGAAATGTTTCTAG

>MpAPT3

ATGACGACGACAGCTGCGGAAAGTGATCCCCGCCTCGCCCACATCAAGGAGTCGATTCGTACCATCCCCGGTTTCCCAAAGGCCGGTATCCTTTTCCGCGATGTGACGACTCTTCTGCTTGATCACAAAGCTTTTAAGGACACCACTGACATTTTTGTCGAACGATACAAGGACATGAAAATAGACGTCGTTGTTGGAATTGAAGCACGAGGTTTCATATTTGGCCCACCGATTGCACTCGCTATTGGAGCGAAATTTGTTCCTCAACGAAAGCCCAAGAAGCTACCAGGCCCTGTTGTCGGTGAGGAGTACGAGTTGGAGTACGGTACAGACCGAATTGAGATGCACGTTGGAGCCGTGCAGCCCGGTGAACGCTGTTTGATTGTGGACGATCTTATTGCAACCGGGGGTACACTTGCTGCAGGAATAAGGCTACTAGAGCGCGTCGGTGCCGAGGTTGTGGAGTGTGCCTGCTTGATCGAGCTTTCGGATCTGAAGGGCCGTGAGAAACTGAAAGGCAAGCCTCTGTTCATCCTGGTAGACTTCGAGGGAGAGTAA

>MpPEX4

ATGGCATCAAGGGCAAGACTGTTCAAAGAGTACAAAGAAGTACAAAGAGACAGAGGGCAGGATTCGGATATTCAGCTTCTTTGTGACGAAACAAACATTTATCGTTGGACTGCACTGATTAAGGGACCAACAGATACTCCTTACCAAGAAGGTGTTTTTCAGCTTGCCATTAATGTGCCGGAACAATATCCTCTAGTACCACCACAAGTTCGTTTCGTGACAAAGATATTTCACCCAAATGTTCACTTCAAGACCGGCGAGATTTGTTTGGACATATTGAAGACAGCTTGGAGTCCGGCATGGACACTGCAGTCAGTTTGCCGTGCTGTTATTGCACTGTTGGCACATCCAGAGGCCGACAGTCCTCTCAATTGCGACTGCGGAAACTTGTTACGATCGGGGGACAATCGGGGTTATCAGTCTATGGCTTTGATGTACACAAGGCTAGCAGCTGTGGCATCCGTGCCAAACAGTTCTGCGTAAG

>MpUBQ10

ATGCAGATCTTCGTGAAGACTCTGACGGGGAAGACCATCACGCTCGAGGTCGAGAGCAGTGATACCATCGACAATGTGAAGGTTAAAATTCAGGACAAGGAGGGAATTCCTCCTGACCAGCAGAGGCTGATCTTCGCCGGCAAGCAACTCGAAGACGGACGCACCCTTGCCGACTACAACATCCAGAAGGAATCCACACTCCATCTGGTGCTTCGGTTGAGGGGAGGGATGCAGATCTTCGTCAAGACTCTGACCGGCAAAACCATCACCCTCGAGGTGGAGAGCTCCGACACTATCGACAACGTCAAAGCCAAGATCCAGGACAAGGAGGGAATTCCCCCAGACCAGCAGAGGCTGATTTTCGCTGGCAAGCAATTGGAAGACGGCCGAACTCTCGCGGACTACAACATCCAAAAGGAGTCGACTCTTCACTTGGTGCTTCGGTTGAGGGGAGGGATGCAAATCTTCGTGAAGACCCTCACGGGCAAGACGATCACTTTGGAGGTTGAAAGCTCCGACACCATCGACAACGTGAAGGCCAAGATCCAGGACAAGGAGGGAATTCCCCCAGACCAGCAGAGGCTGATCTTCGCTGGCAAGCAATTGGAAGACGGCCGAACTCTTGCGGACTACAACATCCAAAAGGAATCAACTCTCCATTTGGTTCTTCGTTTGAGAGGAGGTATGCAGATTTTTGTGAAAACACTCACCGGTAAGACTATCACTTTAGAAGTAGAAAGCTCCGATACCATTGATAACGTCAAAGCCAAAATTCAGGACAAGGAGGGAATTCCTCCAGACCAGCAGAGGCTGATCTTTGCCGGTAAGCAGCTCGAGGACGGCCGAACTCTCGCGGACTACAACATCCAAAAGGAGTCCACTCTTCATTTGGTGCTTCGGTTGAGGGGAGGGATGCAGATCTTCGTCAAGACTCTGACCGGCAAAACCATCACCCTCGAGGTGGAGAGCTCCGACACTATCGACAACGTCAAAGCCAAGATCCAGGACAAGGAGGGAATCCCCCCTGACCAGCAGAGGTTGATCTTCGCCGGTAAGCAATTGGAAGATGGCCGCACCCTCGCAGACTACAACATTCAGAAGGAGTCTACTCTCCATTTGGTGCTTCGTCTGAGGGGAGGTATGCAGATCTTCGTGAAGACCCTCACGGGTAAGACAATTACTCTGGAGGTGGAAAGCTCCGACACCATCGATAATGTCAAGGCTAAGATTCAAGACAAAGAAGGAATTCCCCCAGACCAGCAGAGACTGATCTTCGCCGGCAAGCAGCTCGAAGATGGCCGAACTCTCGCGGACTATAACATCCAAAAGGAGTCCACTCTCCATTTGGTGCTTCGTCTCAGGGGAGGTATGCAAATCTTCGTCAAAACTTTGACTGGAAAGACGATCACTCTTGAGGTAGAAAGCTCGGATACGATCGATAATGTGAAAGCTAAGATTCAGGACAAGGAAGGGATTCCCCCAGACCAGCAGAGGCTGATCTTCGCTGGAAAGCAGCTCGAAGATGGCCGAACTCTCGCGGACTACAACATCCAGAAGGAGTCCACTCTCCATTTGGTGCTGCGTCTCAGGGGAGGTATGCAAATCTTCGTCAAGACTCTGACCGGCAAGACCATCACTCTGGAGGTAGAAAGCTCGGATACGATCGATAATGTGAAGGCCAAGATTCAGGACAAGGAAGGAATCCCCCCTGACCAGCAGAGGTTGATCTTCGCCGGTAAGCAATTGGAAGATGGCCGCACCCTCGCAGACTACAACATTCAGAAGGAGTCTACTCTCCATTTGGTGCTTCGTCTGAGGGGAGGCATGTAA

>MpTUB8

GCGGATACTTTAGCTCCCCGTGAGCGGGCGCTCCAAAGCAGGCCAGCGGCAGAAGAGCTTGTGCAGCTATAAGCTTGGCTGGGCCCGTTCTTTGCTGCGCATCGCACTGCTCGATCTCCGAAAGGATTGAAAGCGGGAGGAAAGTCGCAGTAAAAAAAAAAAAGCTCGATCTCCGAAAGGATTGAAAGCGGGAGGAAAGTCGCAGTAGAAGAGGACGGGGCGGGAAGATATTATCGACGATACTAAACCTGGCAGGGTCGAGGGCGAAGTGGAGGAGGAGGCAGGGAAGGAGGAGAGGAGAGGAGCTGCAGTAGTCTTTCGCTGAGTGCACTTCCTTGAGGTGAAGTCAGCGATCGGAGAGGAGGAGGAGGAGGAGTAGGAGGAGCAGCAGCGGCAGCAGCAGAAGAAGAAGCAGCGGCGGCATTCTGTTCGAGTGTCGCTCGGAGGTCGAAGGGGAATTCTGCCGGACCGATCTTTAAGCTGCAGCAATTTGGACTGCTATTTAATCTTTTTTAGACTTGTTGGCGCATACAGAAAGCAAATCCAAGATCATACCAGTTCGTCAAG**ATG**CGTGAAATTTTACATATTCAGGGTGGACAATGTGGAAACCAGATCGGAGCCAAGTTCTGGGAAGTAGTCTGTGACGAGCATGGTATTGATCCCACTGGGACCTATCAGGGTACCTCCGACTTGCAGTTGGAGCGAGTGAATGTGTATTATAATGAAGCCAGCGGAGGGCGGTACGTCCCTCGCGCGGTTCTCATGGATCTTGAGCCCGGCACCATGGATAGTGTTCGATCAGGTCCTTATGGACAGATCTTTAGACCCGACAACTTCGTCTTTGGGCAAACTGGAGCTGGAAACAATTGGGCTAAGGGGCACTACACCGAAGGGGCTGAACTCATAGACTCTGTTCTCGATGTGGTCCGAAAGGAGGCTGAGAGCTGCGACTGTCTTCAAGGTTTCCAGGTGTGCCATTCGCTTGGTGGAGGAACTGGATCTGGTATGGGTACTCTCCTCATATCTAAAATACGAGAAGAATATCCCGACAGAATGATGCTCACTTTCTCAGTGTTCCCTTCGCCCAAGGTTTCCGACACTGTCGTCGAGCCCTACAACGCTACTCTGTCAGTACATCAGCTCGTAGAGAACGCCGATGAATGCATGGTGCTCGACAACGAGGCTTTGTATGATATCTGC

TTCAGAACGCTGAAGTTGATAACACCTACATTCGGTGACCTGAATCACTTAATCTCCGCGACGATGAGCGGTGTTACCTGCTGTCTCCGATTCCCCGGTCAGCTCAATTCCGATCTCCGGAAGCTCGCCGTCAACCTTATTCCCTTTCCCCGCCTCCATTTTTTCATGGTTGGTTTTGCCCCTTTGACCTCAAGAGGATCCCAACAGTACAGAGCACTTACGGTGCCCGAGCTCACCCAACAGATGTGGGACGCGAAGAACATGATGTGCGCAGCTGACCCGCGCCACGGACGATATCTCACGGCTTCTGCCATGTTCAGAGGGAAAATGAGCACGAAGGAGGTGGATGAGCAAATGATTAACGTTCAAAATAAGAATTCCTCTTACTTTGTCGAATGGATCCCTAACAATGTGAAATCCAGCGTCTGCGACATCCCTCCCACAGGATTAAAAATGTCTTCAACATTTGTTGGGAACTCTACCTCTATTCAAGAAATGTTCAGGCGTGTTAGCGAGCAGTTTACTGCCATGTTTAGGAGAAAAGCTTTCTTGCATTGGTACACAGGAGAGGGCATGGACGAGATGGAATTCACGGAGGCTGAGAGCAACATGAACGATCTGGTATCAGAATATCAGCAATATCAGGATGCCAGCGCAGAGGAAGAACCTGAGTACGAGGATGAGGGTGATGAGGCT**TAG**ATTTTGTGTTTTTTTTTCGAGGCGTCTAAGTCTTAGTTATGTTGACAGGATTTTCTGTCTCTGTAGTCTGTATATTTAACTAATCAGCACTGCAAACTCATGTCTTTTCATTTTCAATAAATAAAGTTGAACCGAGGCCTTTTCCGCTACGAAGCGAGTGATGACGCAAGCCTCCATTTCGGACCCACTTGGTTGGATGAGGCAATCCACCGTCACGAAGGAGAAGCAGG

>MpH3

GAGCTGATATATTTACAACAGAGCTTTAAGCAAAAACGCATCTGTCTGTCCTTGTGGTACGTGGGCAGAGTGGTATGAAGAACTATCCGGGCGGTAGGAAGCAGACTGCAAAGGCGAACTCGTTCCCCTTCGGACCATCAGCAAGGAGATCTTTGTCAGATGAGGAGTCACCATGTGCATTGACTTGTTTCTCTTCAACCGGAATTGATCATGTCCACCAAGTGCTCCAGCTAGCAAAGCTGGGCCTAAGTACCAAGAATCGTTGGACACAGATAGTATGGCATCTACTGAATACCCACCTGGGCCTAAGTACCAAGAATCGTTGGACACAGATAGTATGGCATCTACTGAATACCCACGGGCAGGGGTGTTCCAGCAGAAAATGGAAGCACAGAGATCGCAATTCCTCGACAATAGGCGAGAGGAGATGGCTTAGGCTCGTTCGCCGCGAATTCTTCGAGCGAGCTGAATATCTTTCGGCATAATCGTCACACGCTTGGCATGAATCGCGCAGAGGTTAGTGTCCTCGAAAAGTCCGACGAGGTAAGCCTCGGCCGCTTCTTGAAGGGCCAGGACGGCGCTACTCTGGAACCGCAAATCAGTCTTGAAGTCCTGGGCAATCTCTCGAACCAGCCTTTGGAATGGCAACTTCCTAATCAGAAGCTCAGTGGACTTTTGGTACTTCCTGATCTCGCGCAACGCCACGGTTCCAGGCCTGTATCTGTGAGGCTTCTTCACTCCTCCGGTGGCAGGAGCCGACTTGCGCGCAGCCTTCGTCGCCAGCTGCTTGCGAGGCGCCTTGCCTCCCGTGGACTTGCGAGCTGTCTGCTTCGTTCGAGCCATCTCGAAACACTGGAATTGGAATCGGCCGACGAACACGATGTCGAAATCACAGGGGATGAACGGAACGCTCTAGACTCACGACACTCGAGGGGGACGGAAGGACGTGAGAGTGGGAGATTGGCGAAGAGATTGCTCACGGCAAAGAGAGAACGGGAGAGTGTAAGATTGGTGATTCTGTA

>MpCUL1

ATGACCATGAGTAACGAGCGGAAGGTCATAGAGCTTGAGCAAGGATGGACTTTTATGCAGAAAGGCATTACGAAGCTAAAGAACCTTCTCGAAGGAGTTCCAGAGCAGCAGTTCAACTCCGAAGAATACATCATGTTGTACACTACAATCTACAATATGTGTACGCAGAAACCTCCACAAGACTACTCGCAACAGCTGTACGATAGGTACAAGGAGTCTTTTGAAGAGTACATCAACACTATGGTATTACCCGCGCTTCGAGAGAAACACGATGAATTCATGCTAAGGGAGCTTGTGAAACGATGGGATAACCACAAAATTATGGTTAGGTGGCTTTCTCGTTTCTTCAACTACTTAGACCGCTACTTTATTGCAAGAAGGTCGCTCCCGGCGCTCAATGAAGTGGGCCTCATGTGCTTCCGTGATCAGGTCTATGCCGAAATGAAGAACAATGTCAAGGACGCAGTGATAACTCTGATCGATCGAGAGCGAGAAGGTGAACAAATTGATCGAGCACTGCTGAAGAACGTTCTCGGTATATTTGTGGAAATTGGCATGGGAAACATGGATGCTTACGAGAGTGACTTTGAGATGGCGATGCTTACTGATACTGCTGCTTACTATTCAAGGAAGGCAGCGTCTTGGATTGAGGAGGATTCTTGTCCTGATTACATGCTCAAGGCTGAAGAATGCCTGAAAAGGGAAAAGGAAAGAGTCGGTCATTATCTACACGCTAGCAGTGAACTGAAGTTGCTAGAGAAAGTGCAACACGAACTGCTTTCTCAGTATGAAAATCAGCTATTGGAGAAAGAGCATTCCGGATGCCATGCTCTGTTAAGAGATGACAAGGTGGACGACCTTTCTAGGATGTACCGACTGTTTTGTAGAATTCCAAAAGGTTTGGAACCGGTGGCGGCAATTTTCAGACAGCATGTTACAGACGAGGGAACTGCCTTGGTCAAGCAAGCAGAGGATGCTGCAAGCAATAAAAAGGCTGAGAAAAAGGACACTCATGGAGGCCAAGAGCAGGCATTTGTACGTAAAGTGATTGAACTCCATGACAAGTACTTGCAGTATGTGAACGATTGTTTCATCAACCATTCTCTTTTCCACAAGGCTCTCAAGGAGGCGTTTGAAGTCTTCTGCAACAAAGGTGTAGCAGGAAGTACGAGTGCCGAGTTGCTTGCCACTTTCTGTGAC

AATTTGTTGAAGAAAGGTGGAAGTGAGAAGTTGAGTGATGAAGCTATCGAAGATACTCTTGAAAAGGTCGTCAAGCTCCTTGCTTACATCAGCGACAAGGATCTATTCGCGGAATTTTACAGGAAAAAACTGGCGCGTAGACTTCTGTTTGATAAGAGTGCGAACGATGACCACGAGCGTAGTATTTTGACTAAGCTCAAGCAACAATGCGGTGGACAGTTCACTTCCAAGATGGAGGGCATGGTTACCGACCTGACCCTTGCAAGGGAAAATCAAACGTCTTTCGAAGACTACTTGAGTGAGAACTCAACGTCGAATCCGGGGATTGACCTGACTGTAACTGTCCTTACCACTGGCTTCTGGCCGAGTTACAAGTCTTCAGACCTCGCCTTACCGGCTGAAATGGTTAAGTGTGTTGAAGTTTTCAAGGAATTTTATCAAACGAAAACAAAGCATCGAAAGCTTACTTGGATCTACTCGTTGGGTACATGTAACATCAACGGAAAGTTTGAACCTAAGCCAATCGAGCTCATAGTTACAACCTATCAGGCTGCAGTGCTGCTACTATTTAATGCAGCAGAGAGACTAAGCTATGTCGATATCAAGGGTCAGTTGAATCTCACGGATGAGGATATTGTTAGATTGCTACATTCTTTATCATGTGCCAAACACAAGATTTTGAACAAAGATCCAAACACGAAGTCTGTGGGTTCAACTGACTATTTTGAGTTCAACACCAAATTTACAGACAAAATGAGACGCATCAAGATCCCACTCCCTCCTATGGATGAGAAGAAGAAAGTGATTGAGGATGTGGACAAGGATAGACGATATGCTATTGATGCATCAATTGTTCGGATAATGAAGAGTCGCAAGGTGTTGCCACATCAACAGCTAGTTCTTGAATGTGTTGAGCAGCTTGGCAGGATGTTCAAGCCTGATTTTAAGGTTATCAAAAAACGTATTGAAGACCTTATTGCTCGAGAGTACTTGGAACGTGACAAGGACAATCCCAACATGTTCAGATACCTGGCTTGA

>MpELF5

GTCGTGGCGGGCTCGCGGGCTCTGGCTCGCTCCTCTTCCTCCTGCTCGCTCCCTCCTCTTCGCAAGACGCGGTCGGGGTCCGGCGTCGTCGTCCTCTGGCTTGGGCTCGGGCTCCTCCTCCTCCTCCCTCCTCGGGCCTCCTCTTGTGCTTGCGGAAGAGAGCGAGCTTTTCCTCTGTCTCAGTCTCTTCACAGAGTGAGCCATCGCCAGAGCTTCAAGTGGAGCACAAGGGTTTTCTTTCCATCGCTGCGGCGCCTCCTCATATTATCGGCCTCCAGCAGTAGTGGGGGAGCAGCAGGCATCCAGCAGCGGCTCGCTGTTGTGGGTTATTTTGGAGGATTGCTGATCGGCGCTGAGGGTTGTTGTTCCGCTCCGCCTTGCCCTGCTCTGCTCCGCCCCTACTCGTTGCTGTATTTCGAAGGAGGATTCGGTGTGTGCGCGAGGCAGGTTCCCGGAGCGGTTGAGCAAGCGCTTTCTTCCACCATGTCTGATGATGATCACCACTTCGATTCCAAGGCCGATGCCGGTGCCTCCAAGACCTACCCTCAGCAGGCCGGTACAATTCGAAAGAATGCTTACATTGTGATCAAGCAGAGGCCCTGCAAGGTTGTCGAGGTTTCCACCTCCAAGACCGGCAAGCACGGTCACGCTAAGTGTCACTTTGTGGGTATCGATATTTTCACTGGCAAGAAGCTGGAGGATATCGTTCCTTCCTCCCACAATTGTGATGTCCCTGAGGTCGTTCGTACCGACTACCAGCTGATTGACATATCCGAGGATGGATTCGTGTCCCTCCTGACTGAGTCTGGTGATACCAAGGATGACCTTCGTCTCCCCACCGACGACCAGCTCCTCACCCAGATCAAGGATGGATTTGGCGACGGAAAGGACCTTGTTGTGACAGTGATGTCCGCCATGGGAGAGGAGCAGATCTGTGCTCTCAAAGATATCGGTCCTAAGAACTAG

>MpGAPC1

TGGCACTATTATCTGCCTCCACACCTGCTCTTCCAGCTGCTACTTCAATTGGCGTTCTCTGCTCTCAGTCCATCAGCTCTTCTCTCCCGTTCGAGTGTTACAGCTCCTGCGAATTGACTCGTACCTCGTATTTATTCATCGATCGGATCACTGAAGCTTCGATCAATCTTCTTTCCTTACGTACAAGCGGGATACTCAAGCACCTTCCCATCATTCTTCTGGTCGGACCAGAGTTTTGTTTTTGACGGTTTTGCAGTCGCAGTCTCGAAACC**ATG**GCAGGATCAACTGAGCCATCGACGATCAGCTCAGGAAAAGTGAAGATCGGAATCAATGGCTTTGGAAGGATCGGACGACTGGTGGCGCGTGTCGCCTTGGAACGTGATGACATCGAGTTGGTTGCCGTGAACGATCCTTTCATCAGCACCGAATATATGGCCTACATGTTCAAATACGACAGTGTGCACGGACGGTATACCAAGAACACCATCGAAGCCAAGGACGACAAGACTCTTCTTTTTGGAAAGTTCCCTGTCTCCATCTTCCAGTGCCGCGACCCAGCTGAAATCCCCTGGGGCCAAACCGGAGCCGAGTACGTCGTCGAGTCTACCGGTGTGTTCACCACCAAGGACAAGGCCGCTGCCCATCTCAAGGGCGGAGCGAAGAAAGTCGTCATCTCGGCTCCGAGCGCAGATGCTCCAATGTTCGTCATGGGAGTGAACGAGAAGGAATATACCACTGACATTGATATTGTGTCCAACGCAAGTTGCACCACCAACTGTCTGGCCCCTCTTGCCAAGGTTATTCACGATAAATTTGGAATCGTCGAAGGTCTCATGACAACGGTGCACTCAATTACAGCAACTCAGAAGACCGTCGATGGGCCATCTGCGAAGGACTGGAGAGGTGGACGTGGTGCTAGCTTCAACATCATTCCCAGCTCCACGGGAGCCGCAAAGGCCGTAGGCAAGGTGTTGCCTGCTTTGAACGGGAAGCTGACTGGAATGTCATTCAGAGTACCCACCGCTGACGTTTCAGTCGTCGATCTCACCGTCCGAACCGAGAAGAAGGCAACGTACGAAGAAGTCAAGGCCGCTATCAAAGCTGAATCTGAAGGCGAAATGAAGGGAATCATGGGCTACACCGAGGACGATGTTGTGTCCACCGATTTCATCGGTGATAGCAGGTCGAGCATCTTCGACG

CCAAGGCCGGAATTGCTCTGAACGACAACTTCATGAAGTTGGTATCTTGGTACGACAACGAATGGGGCTACAGCAACCGAGTGGTGGACCTGATCTTGCATATGGCGTCTACCCAG**TGA**GAACCAAATTCTGTTCTTTCTACTCCCACTACTCTTGTAATCTTTGCAGCCGTTCTGTCCGCAAGTCGGAAAGTGGCCATTTTTGCACTGTTCTACACGATGATTGTTGGAGTAGGCAAGTTTGTTGTTCTGGTAGAGAGTATCCATCGAAAGTGGATATTTTGGTGACACTGGCACTCACCTGGACTTGTCATATTCGACATGATTCATGTATAGTTTTTGTTTTATAAATAAAAGAATAAGTACACGGGAAACGAAATCCTTGAAATTTTCCTCAACATCAAATCACAATTATAAGCCTTTTTGTCAAGACTTCCCATATATTTTTATTAGGAAATTGTATTTGATAATTCTATGTTCAATACACCCACACAGCATGATTGATATACAGAGTTAAATCTCCCAATAGTGACGCACGTTGATGATAGAGAAGCTTGTTTTTTTTTTTTTTTTTTTTTTTTTTTTTTTTTTTTTTTTTTGGGAGATGTCACTCACAAGATTTTTGAGTGACAAAGTGTAACCTTTGAGTGTAAACTCCCAATAGTAGTAATATACAAGGATGTTAGGGTTGGATATCATGATAGGCG

>MpPHT1

ATGTCAGGCGAATCAGCTGTTGGAATGGAGGTGTTGAAGACCCTCGACAAAGCCAAGACTCAGGCCTACCATTTTAAATCGATAGTGATTGCAGGAATGGGATTTTTCACCGACGCTTATGATCTCTTTTCGATCTCCACAGTCACTAGACTGCTGGGTAGGCTGTATTACTACGATCCTGCACTCGGCAAGCCTGGAACTCTGCCACCGAATGTTGCAGCAGCAGTCAATGGAGTGGCTTTTGTAGGCACGCTGCTCGGGCAGCTCTTCTTCGGATGGGCAGGTGACAAATTCGGGCGTAAAAAGGTTTATGGGATCACTCTTGTGCTTATGATTATCACGTCCATTGCGTGTGGACTCTCTCTGGGCAGCAACGCCACCAGTGTCATGACCACCCTGTGCTTCTTCCGCTTCTGGCTGGGATTCGGAATCGGGGGAGATTACCCCCTCTCGGCCACAATCATGTCCGAGTACTCCAATACCAAAACTCGGGGAGCATTCATCGGAGCAGTGTTCGCGATGCAAGGGTTCGGAATCCTCTCCGGAGCCACAGTTGCCATCATTCTCTCTGCAGCCTTCAATGCTGCTTATCATCGACCAGCATTTCAAGTCGATCAAATCAGGTCCACCCCTCCTCAGGCGGACTACGTGTGGCGTCTACTCTTCATGTTCGGTGCCCTTCCGGCTGCGCTGACCTACTACTACCGAATCCGAATGCCCGAGACTGCTCGGTACACGGCTCTGGTCGAGAAGAACAACGACCAGGCCGCCAGAGATATGGAGAAGGTGCTGCACGTCCAATTCCAAACCAAGCATGACGATTCTTCAGTCGCGATGGGCGTGCAGAAACATCAAGCGGCAGCGGAACGTGAAGCTGACAAGAAAGTGCAGTACAAACTGTTCAGTCACGAGTTTCTCAGACGCCATGGTTATCAGCTGCTGGGATGCACAGTGTGCTGGTTCATGCTGGATGTGGCTTTCTACAGCCAGAATCTGTTCCAGAAAGACATCTTCAGCGCCGTGGGGTGGATTCCAGCAGCCAAGAAAATGAGCGCCCTGGAGGAGGTGTTCAAGATTAGCCGAGCTCAAGCTTTGATTGCCCTTGTATCCACCGTGCCTGGCTACTGGGTGACAGTTGCTCTCATCGACAGAATTGGAAGGTGGTGGATTCAGCTGAATGGCTTCTTCATGATGACACTATTTATGCTCGTCTTGACCTTCGACTACTACAACCTCAGGGGAGATCCATGTCCCGCCGACTCATCCAAGTACTGCGGTGGTAACCATTTAGCGTTTATTGCCATGTACGCTCTGACCTTCTTCTTTGCCAACTTCGGGCCCAACTCCACAACATTCATCGTGCCGGCGGAGCTTTTCCCCGCCAGGCTAAGGTCTACTTGTCACGGAATATCCGCAGCATCGGGCAAAGCTGGAGCTATAGTCGGGGCATTTGGATTCCTATATGCTAGCCAGAGCCAGTATGCCGGTAAGCAGGATTATGGTTATCCCAACGGAATCGGTATTAAGAATTCTCTGCTAGTACTTGCTATCTGCAATGCCATAGGTTTCTTCTTCACGTTCTTCGTTCCGGAAACGAAGGGTAAGTCTTTGGAGGAACTTTCTGGGGAGAACGAAGAGGACACTAACGCAACTGAGGTGAGAGAATAA

>MpNRT2

ATGCCTCTGGTGTCCACTCCGGTGGGATTCATCCTGGTGCGATTCTTCATCGGTTTCTGCCTGGCGACTTTCGTGTCTTGTCAGTTCTGGATGAGCTCCATGTTCAACAGCAAAATCGTGGGAATCGCTAACGGCACGGCAGCCGGATGGGGGAACATGGGAGGAGGCATCGTTCAGCTGGTGATGCCCCTGGTGTTCGACATCATCCGCCGAGACATCGGGTCAGAGAAGTTCACTGCTTGGAGGATCGCCTTCTTCGTCCCCGGCGTGCTGCAAGTGATGATGGGACTGCTCGTCTTGACCCTGGGCCAAGATTTGCCTGATGGAAACTACGCCGAGCTAAAAAGAGAGGGCGAGAAAGTCAACGACAGCTTCAAAAAGGTATTTTTGTACGCTGTCACCAACTACCGCACGTACATTTTTGCTCTGACCTACGGCTATTGCTTCGGAGTGGAGCTCACAGTCGACAACATCATCGCCGAGTACTTCTACGATCGCTTCGACCTGAACCTGCAAACTGCGGGAATCATCGCATCCACATTCGGGCTCATGAACTTGTTCTCTCGCCCTCTGGGAGGAGTCCTGTCTGACGTTGTGGCCGCGAGGTGGGGAATGAGAGGTCGCCTCTGGAATCTGTGGATCATCCAAACCGTGGGAGGAATTCTGTGCATCGTTCTGGGACTCACGGGTCAGCTAGGACCTGCCATCGCCGTCATGCTCATCTTCTCGTTCTTCGTCCAGGCAGCTTGCGGAGTGGGAGGAGTCCTCTCCGACGTGGTGGCTGCGAGGTGGGGAATGAGGGGCCGCCTCTGGAACTTATGGATCATCCAGACTGTGGGAGGAGCTTTGTGCATCGCCTTGGGTCTGACGGGTCAGCTGGGACCTGCCATCGCCGTCATGTTGGTTTTCTCGTTCTTCGTCCAGGCAGCTTGCGGGGCCACTTTCGGCATCATCCCCTTCATCTCCCGAAGGTCTTTGGGAATCATCTCTGGCGCCACTGGAGCTGGTGGAAACATCGGAGCCGTGACCACTCAGGGAATCTTCTTCATGAGCTCCAAGTACTCGACGGAGAACGGAATCATGCTCATGGGCATCATGATCGTTGCCTGCACCCTGCCAATTATCTTCGTCCACTTTCCCCAGTGGGGAAGCATGCTCTTCCCCCCATCCACGGCCACCGAGGAAGATTATTACGTCTCAGAGTGGAGCAAGGAAGAGCAAGCCAATGGTCTTCATAACGCTAGCATGAAATTCGCCGAAAATGCCAAGTCCGAGAGAGGGCGACAGTCGCCCCCCAGGTCAAAGTCAGATAATGTTGGTGCGGACCAGCTCTGA
